# Combgap Promotes Ovarian Niche Development and Chromatin Association of EcR-Binding Regions in *BR-C*

**DOI:** 10.1371/journal.pgen.1006330

**Published:** 2016-11-15

**Authors:** Anna Hitrik, Malka Popliker, Dana Gancz, Zohar Mukamel, Aviezer Lifshitz, Omer Schwartzman, Amos Tanay, Lilach Gilboa

**Affiliations:** 1 Department of Biological Regulation, Weizmann Institute of Science, Rehovot, Israel; 2 Department of Computer Science and Applied Mathematics, Weizmann Institute of Science, Rehovot, Israel; 3 Mol. Genetics and Biochemistry, Faculty of Medicine, Tel Aviv University, Tel Aviv, Israel; 4 Childhood Leukemia Research Institute, Sheba Medical Center, Ramat Gan, Israel; Stanford University School of Medicine, UNITED STATES

## Abstract

The development of niches for tissue-specific stem cells is an important aspect of stem cell biology. Determination of niche size and niche numbers during organogenesis involves precise control of gene expression. How this is achieved in the context of a complex chromatin landscape is largely unknown. Here we show that the nuclear protein Combgap (Cg) supports correct ovarian niche formation in Drosophila by controlling ecdysone-Receptor (EcR)- mediated transcription and long-range chromatin contacts in the *broad* locus (*BR-C*). Both *cg* and *BR-C* promote ovarian growth and the development of niches for germ line stem cells. BR-C levels were lower when Combgap was either reduced or over-expressed, indicating an intricate regulation of the *BR-C* locus by Combgap. Polytene chromosome stains showed that Cg co-localizes with EcR, the major regulator of *BR-C*, at the *BR-C* locus and that EcR binding to chromatin was sensitive to changes in Cg levels. Proximity ligation assay indicated that the two proteins could reside in the same complex. Finally, chromatin conformation analysis revealed that EcR-bound regions within *BR-C*, which span ~30 KBs, contacted each other. Significantly, these contacts were stabilized in an ecdysone- and Combgap-dependent manner. Together, these results highlight Combgap as a novel regulator of chromatin structure that promotes transcription of ecdysone target genes and ovarian niche formation.

## Author Summary

Germ line stem cells (GSCs) supply either eggs or sperm throughout the life- time of many organisms, including mammals. For their function, GSCs require input from somatic niche cells. Understanding how niches form during development is an important initial step in understanding how stem cell units form, and by extension, how they may regenerate. In this work we describe a new function for the chromatin binding protein Combgap in ovarian niche formation of the model organism *Drosophila melanogaster*. Combgap is required for the correct expression of another factor, BR-C, in somatic ovarian cells. *BR-C* is one of the central target genes of the steroid hormone ecdysone, and its expression is controlled by the ecdysone receptor (EcR). Interestingly, EcR-enriched regions within the *BR-C* locus are engaged in long-range contacts that are stabilized by ecdysone in a Combgap-depended manner. We also found that EcR binding to chromatin depends on WT levels of Combgap. BR-C regulates GSC unit establishment, intestinal stem cells, immune responses, and many other processes. Understanding Combgaps’ function in shaping the *BR-C* chromatin landscape is a first step towards better appreciation of how this important locus is controlled, and the general machinery coupling gene expression to 3D chromatin structure.

## Introduction

The normal function of many adult organs depends on stem cells and their niches, which make functional and structural units. To make these units, niche precursors and stem cell precursors must coordinate their development. Understanding how this occurs is key to understanding organogenesis and regeneration. Here we show that in Drosophila, the chromatin binding protein Combgap (Cg) controls proliferation and differentiation of somatic niche and germline stem cell precursors, thereby promoting correct ovarian stem cell unit formation.

*cg* mutants were first isolated by Calvin Bridges in the 1930s [1]. The Cg protein contains 11 putative C2H2 zinc finger domains, a Sir2 domain, a Lambda-1 domain and a poly-glutamine stretch. These motifs suggest Cg acts as a chromatin-binding factor that can either promote or repress transcription. Indeed, Cg has been previously shown to control gene expression in several organs. In the visual cortex, Cg represses the Wingless target genes *optomotor blind*, *decapentaplegic* and *aristaless* [2]. In leg and wing discs, Cg represses *cubitus interruptus* (*ci*) expression in the posterior compartment and promotes *ci* expression in the anterior compartment [3,4]. The pleiotropic effects of Cg and its association with several major signaling pathways suggest that this nuclear protein performs a general function for gene transcription. We now show that Cg is required for correct transcription from the *broad complex* locus (*BR-C*), which is one of the major genes required for Germ Line Stem Cell (GSC) unit formation in the developing ovary of *Drosophila melanogaster*.

**Fig 1:**
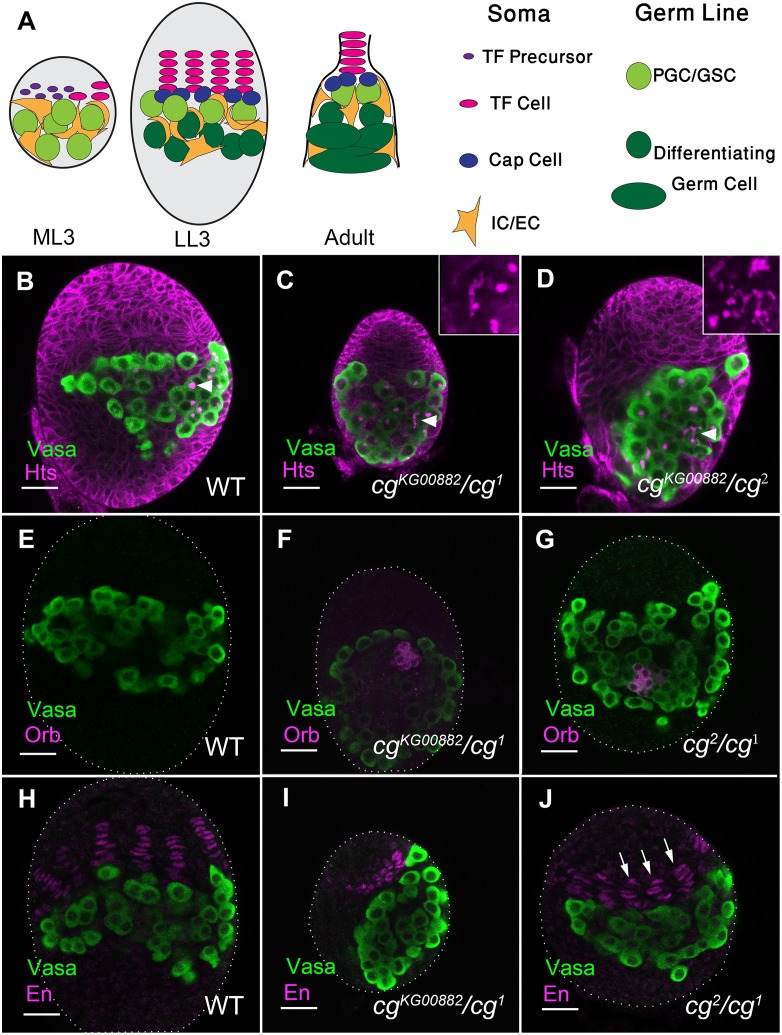
Cg is required for correct ovarian development. (A) An illustration of mid larval 3^rd^ instar (ML3), late larval 3^rd^ instar (LL3) ovary and an adult germarium. Cell types are defined. (B-J) Anti-Vasa (green) marks PGCs. (B-D) Anti-Hts outlines somatic cells and fusomes within germ cells (magenta). (B) WT LL3 ovaries, PGCs contain spherical fusomes (arrowhead), n = 20. (C, D) 48% of cg^1^/ *cg*^*KG00882*^ (C, n = 28), 67% of *cg*^*KG00882*^/*cg*^*2*^ (D, n = 18) and 47% of *cg*^*1*^*/cg*^*2*^ (not shown, n = 19) mutant ovaries contain cysts with branched fusomes (insets). (E-G) Anti-Orb (magenta) marks 8- and 16-germline cysts. (E) WT PGCs are devoid of Orb at LL3 (100%, n = 20). (F, G) 11% of *cg*^*1*^*/cg*^*KG00882*^ (F, n = 17), and 23% of *cg*^*1*^/*cg*^*2*^ (n = 11) mutant ovaries contain Orb-positive cysts. (H-J) Anti-En marks TF cells (magenta). (H) WT ovaries contain well-formed TFs. (I, J) *cg*-mutant ovaries contain TF cells that have not yet organized into filaments (I) or shorter filaments (J, arrows). n and p values are presented in Table 1. Bars are 20 μm.

The Drosophila ovary makes a good model with which to study stem cell unit formation. During early larval development both Primordial Germ Cells (PGCs, the precursors of GSCs) and somatic cells proliferate [5,6,7]. Somatic niche formation initiates when a pool of proliferating somatic cells become non-proliferating Terminal Filament (TF) precursor cells (Fig 1A). TF precursors accumulate throughout third instar, acquire adult TF markers from mid-larval third instar (ML3), and then form filaments by convergent extension [8]. By Late Larval third instar (LL3) all 16–20 TF stacks, which are contained within a single ovary, have formed [9,10,11,12]. Following somatic niche formation, PGCs initiate their differentiation. However, PGCs that are close to the newly formed niches are spared and become the adult GSCs [6,7,13,14].

**Table 1:** Combgap affects ovarian size and niche numbers.

Genotype	TF stack number ± stdev (n)	t-test p-value	Size (% of WT) ± stdev (n)	t-test p-value
cg*/β-Gal	19.51 ± 2.89 (40)	--	100 (34)	--
*cg*^*2*^*/cg*^*KG00882*^	16.42± 4.05 (24)	0.0008	77.3±20.3 (36)	3.39e-7
*cg*^*1*^*/cg*^*2*^	16.73 ± 3.65 (42)	3.24e-5	85±19 (37)	0.0001
*tj*-Gal4, *cg*^*1*^*/ cg*^*KG00882*^	0.92 ± 1.16 (25)	7.14e-32	37 ± 1.76 (29)	4.44e-35
*tj*-Gal4, cg^1^/ cg^KG00882^; *UAS*-*Br-Z2*/+	4.2 ± 2.11 (59)	3.7e-14**	46.5 ± 2.0 (47)	1.7e-5**
*tj-*Gal4 / UAS-β-Gal	19.11±2.33 (61)	0.19	102±15.3 (52)	0.34
*tj*-Gal4; *UAS*-*cg*^*HMS01145*^	17.25 ± 2.54 (16)	0.059	105 ± 13.67 (16)	0.065
*tj*-Gal4 / *cg*-GFP; *UAS*-*GFP*^*RNAi*^	13.84 ± 5.59 (13)	4.76e-5	73.5 ± 28 (13)	0.006
*tj*-Gal4; *cg*^*LA00629*^	3.33 ± 4.3 (27)	1.12e-14	34.8 ± 8.4 (25)	4.15e-27

* Either cg^1^/β-Gal or cg^KG00882^/β-Gal were used as controls.

** t-test p value was determined compared to *tj*-Gal4, *cg*^*KG00882*^/*cg*^*1*^; +/+ ovaries.

Our previous work underlined the importance of the hormone ecdysone as a switch signal between proliferation and differentiation and as a coordinator of somatic niche formation with GSC establishment [7,8,13]. At early larval stages, the ecdysone receptors EcR and Ultraspiracle (Usp) act as repressors of niche and PGC differentiation. This allows PGCs and somatic precursors time to proliferate. At third instar, consecutive ecdysone peaks promote niche formation and then PGC differentiation. Ecdysone signaling is known to activate many genes. One of its earliest targets is the *BR-C* Locus [15]. This locus encodes four related transcription factors that share a BTB domain and differ in their zinc finger moiety (Br-Z1, Z2, Z3 and Z4) [16,17,18]. In larval ovaries, ecdysone activation is associated with up-regulation of Br-Z1 [13]. *BR-C* is a large locus (over 100KBs) that contains many control elements, including two experimentally confirmed proximal and distal promoters [16,17]. In addition to its major role in GSC unit formation, *BR-C* transcripts are required for the correct development of Intestinal stem cells, imaginal discs, and the nervous system [19,20,21,22]. It also functions during immune response, metamorphosis and developmentally regulated cell death [18,22,23]. Understanding how transcription is controlled in this complicated locus will therefore provide us with a deeper insight into the transcriptional control of many developmentally regulated processes.

Here we show that ovarian development depends on Cg and its control of *BR-C* expression. Furthermore, we show that two EcR-enriched regions in *BR-C* are associated with each other and that this association increases in an ecdysone- and Combgap-dependent manner. We propose that Cg may act as part of a general machinery that modifies high-order chromatin associations.

## Results

### Cg promotes niche formation and represses precocious PGC differentiation

In a screen to identify novel regulators of ovarian development [13] we identified the transcription factor Combgap (Cg) as required for proper niche formation and PGC maintenance. To follow the differentiation status of PGCs, we used anti-Hts antibodies that label the fusome, an intracellular organelle within germ cells. In wild type LL3 ovaries, PGCs do not yet differentiate to form germline cysts, and each PGC carries a spherical fusome (Fig 1B, arrowhead) [6,24,25]. By contrast, ~50% of *cg*-mutant gonads from three different allelic combinations contained branched fusomes, suggesting PGCs differentiated precociously (Fig 1C and 1D, arrowheads, insets, for quantification see legend). Anti-Orb antibody, which robustly stains more developed 8- and 16 cell cysts [26], confirmed these observations. No Orb labeling was observed in WT ovaries (Fig 1E). However, Orb was expressed in *cg*-mutant gonads (Fig 1F and 1G). Thus, both fusome morphology and Orb expression demonstrate that the wild-type function of Cg is required to prevent precocious PGC differentiation.

In addition to PGC maintenance, Cg also affected niche formation. To follow TF cell accumulation and stack formation we used anti-Engrailed (En), which specifically stains TF cells [27]. At LL3, TF cells in WT or heterozygous ovaries were already stacked to form the adult number of niches (Fig 1H, Table 1) [8,12,28]. By contrast, TF formation was defective in *cg* mutants. This was particularly noted in the *cg*^*1*^*/cg*^*KG00882*^ combination (Fig 1I, Table 1). However, fewer TFs were also noted in other allelic combinations (Table 1). These ovaries also displayed shorter TFs (Fig 1J, arrows), an additional indication for a difficulty in TF formation. These phenotypes suggest that Cg is required for the proliferation/survival of somatic precursors, for the process of TF specification or for its correct timing [7].

In accordance with a possible role for Cg in precursor cell proliferation, *cg*-mutant gonads were significantly smaller than WT (Table 1). Vital dye staining (Propidium Iodide, PI) indicated that cell death did not increase in *cg*-mutant ovaries (of 18 *cg*^*1*^*/cg*^*KG00882*^ ovaries, 16 were not labeled by PI at all, and 2 had 1–3 dead cells, a similar level to WT ovaries)[8]. On the other hand, phospho-Histone H3 (pH3) staining, which marks mitotic cells, was significantly decreased in *cg*-mutants (0.15±0.03 pH3-positive cells per 1 μm^3^ in WT ovarian volume, n = 21, as compared to 0.08±0.02 per 1 μm^3^ in *cg*^*1*^*/cg*^*KG00882*^ ovaries, n = 14, t-test p-value 1.35e-6). Thus, Cg affects gonadal size by promoting cell proliferation but not cell survival.

### Cg is required in the soma for ovarian development

**Fig 2:**
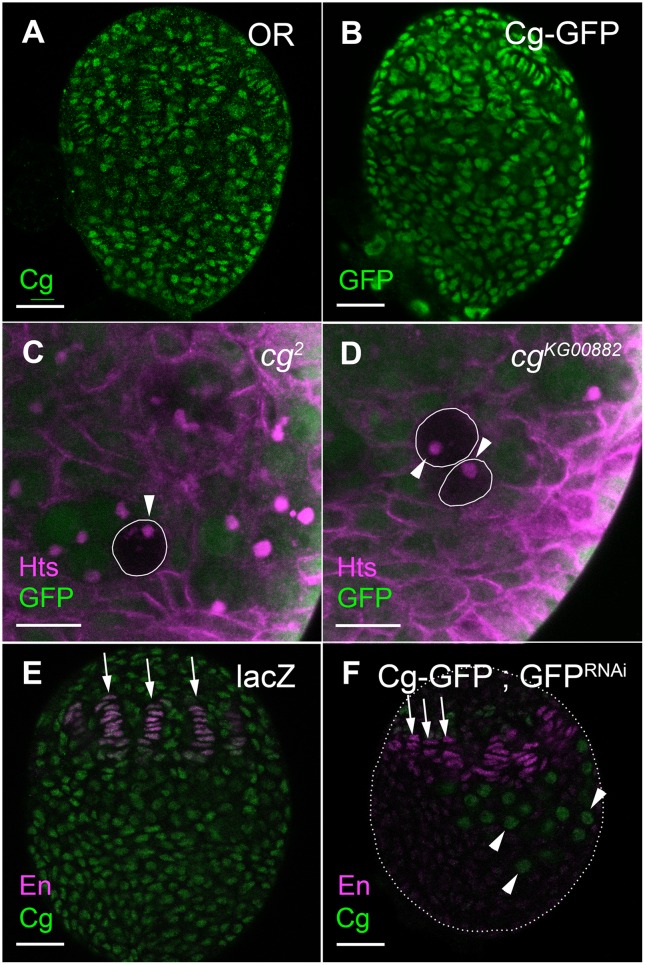
Cg is required in somatic cells for gonad morphogenesis. (A, B) Both somatic and germline nuclei are stained by anti-Cg (A, green n = 30) or anti-GFP (B, green, n = 28). (C, D) Anti-GFP (green) marks WT cells. Anti-Hts (magenta) outlines somatic cells and marks fusomes in PGCs. *cg*^*2*^ (C, n = 52) or *cg*^*KG00882*^ (D, n = 21) mutant PGCs remain undifferentiated and harbor spherical fusomes (arrowheads). (E, F) Anti-Cg is in green and TFs are marked by anti-En (magenta). (E) In control LL3 ovaries, all nuclei express Cg and TFs are long and well formed (arrows). (F) Cg-GFP ovaries. Cg is reduced by *tj*-Gal4 driving GFP^RNAi^ in somatic cells. Cg is still expressed in germ cells (arrowheads) and a few anterior somatic nuclei. Ovaries are smaller and contain less TFs (quantification in Table 1). In addition, many TFs are shorter than WT TFs (compare arrows in 2E, 2F), indicating less developed TFs. Bars in A, B, E, F are 20 μm, and in C, D are 10 μm.

Cg promotes somatic development and niche formation on the one hand, and represses precocious PGC differentiation on the other. To determine how might Cg affect both soma and germline, we first examined its expression using anti-Cg antibodies [4], and found that it was present in all nuclei of WT LL3 ovaries (Fig 2A). Similar results were obtained by examining the expression of a GFP protein trap inserted in the Cg locus (Fig 2B)[29]. The ubiquitous ovarian Cg expression is in accord with its ubiquitous expression in other organs [2,4].

To determine the origin of the somatic and germline phenotypes of *cg*-mutants, we used mosaic analysis. Germline clones of either *cg*^*2*^ or *cg*^*KG00882*^ did not develop as cysts within larval ovaries, and contained spherical fusomes (Fig 2C and 2D). Similarly, depletion of Cg specifically from PGCs by expressing *UAS*-*cg*^*RNAi*^ with *nos*-Gal4 resulted in normal development of both somatic and germline lineages (S1 Fig). We conclude that Cg does not act within PGCs to maintain normal ovarian development.

Expression of *UAS-cg*^*RNAi*^ with *tj*-Gal4, which is specific to the ovarian soma, also failed to produce phenotypes, possibly due to a low efficiency of the RNAi (S1 Fig, Table 1). Indeed, expression of an efficient RNAi line against GFP in Cg-GFP ovaries resulted in low Cg levels in most somatic ovarian nuclei (Compare Fig 2E and 2F and S1 Fig). GFP^RNAi^ ovaries were significantly smaller and contained fewer TFs (Table 1). In addition, the formed TFs in the GFP^RNAi^ ovaries were less developed and incorporated fewer TF cells per TF stack (Compare TFs under arrows in 2E, 2F, S1 and S2 videos). Thus, Cg reduction in the ovarian soma can phenocopy the somatic ovarian phenotypes of *cg* mutants. By contrast, PGC development was normal in the GFP^RNAi^ ovaries (S1 Fig). One possibility is that PGC differentiation requires simultaneous depletion of Cg in both somatic and PGC nuclei. Alternatively, systemic effects could be causing PGC differentiation in *cg* mutant animals (see below).

### Cg is required for the correct expression of Broad-Complex

An additional phenotype of *cg*-mutant animals was their failure to pupariate at the end of larval development. *cg*-mutant larvae continued feeding and growing for several days after their WT siblings pupariated, eventually dying as giant larvae (S2 Fig). Such phenotypes suggest that Cg may somehow affect the ecdysone pathway, which induces molting and pupariation [30]. Since ecdysone signaling is required for ovarian development [8,13,31,32], we investigated whether Cg affected the ovarian ecdysone response, and in particular the Broad-complex (BR-C), its major ovarian target during larval development [8,13].

**Fig 3:**
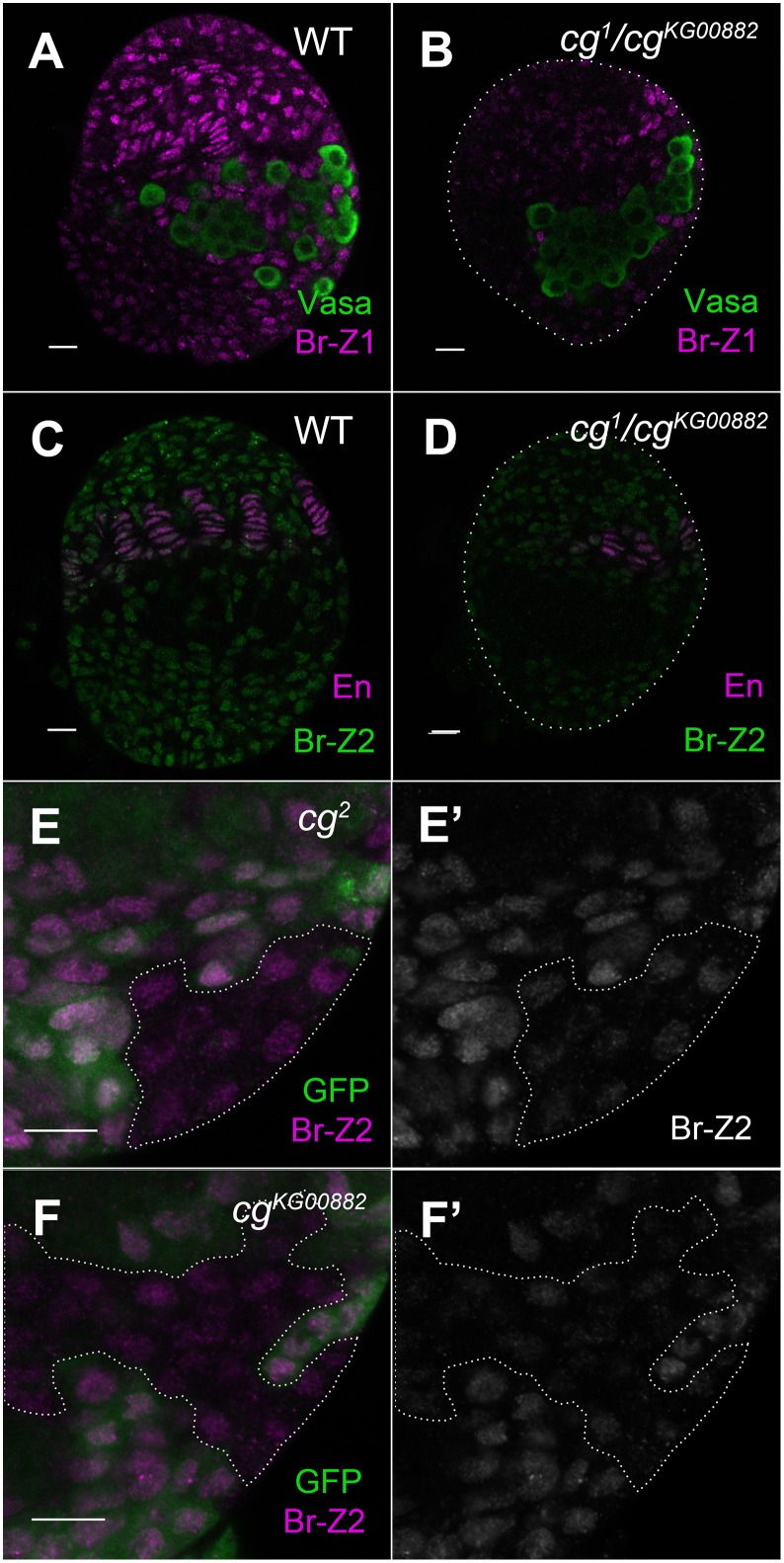
Reduced Broad expression in *cg* mutant ovaries. (A, B) Ovaries were imaged at the same time with the same confocal settings. Germ cells are labeled by anti-Vasa (green). (A) At LL3, all WT somatic nuclei express Br-Z1 (magenta). (B) Reduced Br-Z1 expression in *cg*-mutant ovaries. (C, D) Ovaries were imaged at the same time with the same confocal settings. TF cells are labeled by anti-En (magenta). (C) At LL3, all wild type somatic nuclei express Br-Z2 (green). (D) Reduced Br-Z2 expression in *cg*-mutant ovaries. (E-F’) Mosaic analysis of Br-Z2 expression in *cg*-mutant cells. GFP (anti-GFP, green) marks wild type cells. *cg*-mutant clones are outlined. (E, E’) *cg*^*2*^ mutant cells express lower Br-Z2 levels (magenta in E, grayscale in E’). (F, F’) *cg*^*KG00882*^ mutant cells express lower Br-Z2 levels (magenta in F, grayscale in F’). Bars are 10 μm each.

Of the four Br isoforms, Br-Z3 is not expressed in the ovary and Br-Z4 is expressed at very low levels at the larval-pupal transition, after gonad morphogenesis has largely occurred. We therefore concentrated our analysis on Br-Z2, which is expressed in the somatic ovary from early larval stages, and on Br-Z1, which is induced by ecdysone at mid-3^rd^ instar [13]. Isoform-specific antibody staining in *cg*^*1*^/*cg*^*KG00882*^ ovaries revealed a reduction in Br-Z1 and Br-Z2 protein levels (For Br-Z1, n = 13, t-test p-value 1.44e-7, Fig 3A and 3B. For Br-Z2, n = 19, t-test p-value 2.13e-10, Fig 3C and 3D). Since BR-C levels could be indirectly affected by the retarded development of *cg*-mutant animals, we also analyzed Br-Z1 and Br-Z2 in somatic mutant clones that were generated in a WT heterozygous background. Interestingly, *cg-*mutant somatic clones showed no reduction of Br-Z1 (S3 Fig), suggesting that gonadal Br-Z1 expression could be indirectly affected by the developmental retardation of *cg* mutants. By contrast, Br-Z2 levels were consistently reduced in *cg* somatic clones (Fig 3E–3F’). This demonstrates that Cg promotes Br-Z2 expression in a cell-autonomous manner, which is independent of changes in larval physiology or development.

To determine the extent to which reduced Br-Z2 expression contributes to gonadal phenotypes, we over-expressed Br-Z2 in *cg*^*1*^/*cg*^*KG00882*^ ovaries using a UAS promoter. Br-Z2 gonadal expression was rescued in these animals, and gonadal size and TF numbers increased, as compared to *cg*^*1*^/*cg*^*KG00882*^ mutants (Table 1, S4 Fig). However, gonads were not restored to WT state, suggesting that this pleiotropic gene affects ovarian development via multiple changes in gene expression.

### Cg over-expression represses Broad expression and blocks ovarian development

**Fig 4:**
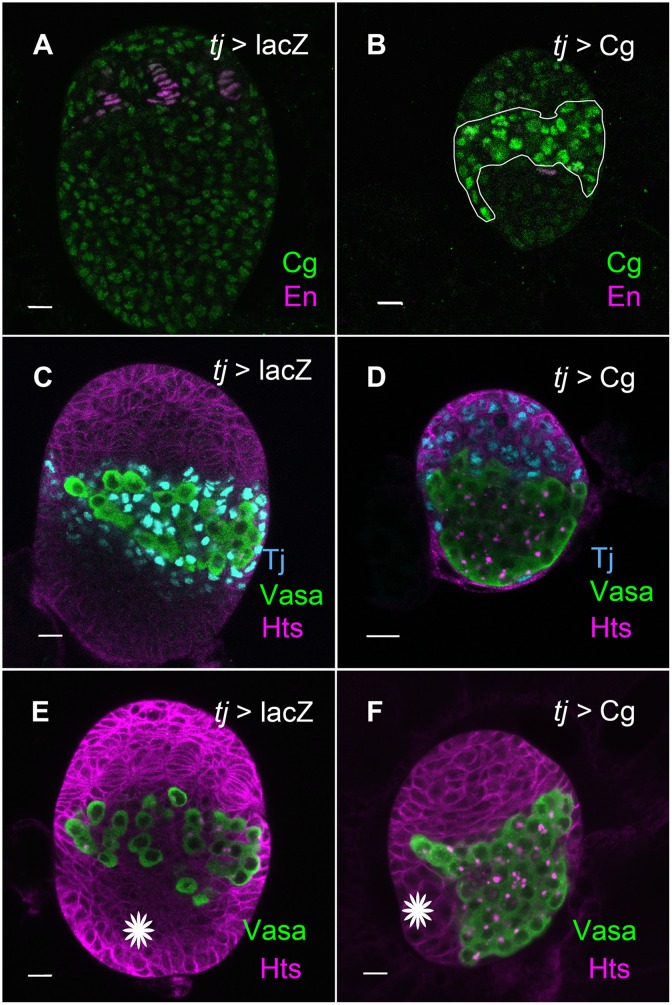
Over-expression of Cg disrupts ovarian morphogenesis. (A, B) LL3 ovaries, Anti-Cg is in green and anti-En marks TFs (magenta). Images were taken at the same day and in the same confocal settings. (A) In wild-type ovaries, TF stacks are completely organized and all somatic nuclei express uniform Cg levels. (B) Over-expression of Cg (outlined) results in extremely small ovaries containing few TFs. (C-F) PGCs are marked by anti-Vasa (green), anti-Hts (magenta) outlines somatic cells and fusomes within PGCs. (C, D) LL3 ovaries, anti-Tj marks ICs in Cyan. (C) In the wild type, ICs intermingle with PGCs. (D) In Cg-over-expressing ovaries, no ICs are present between PGCs. Tj is expressed in all somatic cells. (E) PGCs occupy the middle of the ovary and are interspaced with ICs. Swarm cells are at the posterior (white star, bottom) of the ovary. (F) Cg-over expressing ovary. PGCs are grouped together without ICs to separate them. Swarm cells, which failed to migrate to the posterior, are located medially (white star at the side). Bars are 10 μm each.

To further probe how Cg might affect BR-C expression, we over-expressed it using a UAS- insertion line upstream of *cg*. Cg over-expressing LL3 ovaries were markedly smaller than WT ovaries and contained very few TFs (Fig 4A and 4B and Table 1). To further determine the developmental stage of Cg over-expressing ovaries, we stained them with anti-Tj. In young WT ovaries, Tj is expressed in all somatic gonadal nuclei, but during third instar, Tj expression is gradually restricted to ICs [5,33]. Indeed, only ICs were labeled by anti-Tj in WT LL3 ovaries (Fig 4C). By contrast, all somatic cells were labeled in the Cg over-expressing gonads, and none of these cells intermingled with germ cells (Fig 4D). Thus, Cg-over expressing ovaries seem arrested at a younger developmental stage. Another feature that characterized both Cg-over expressing ovaries and young ovaries, was the absence of a posterior group of somatic cells (swarm cells), which migrate to the posterior during third instar. In Cg-over expressing ovaries, swarm cells failed to migrate and remained at the medial side (Compare Fig 4E to 4F, white star). Combined, these features suggest that Cg-over expressing ovaries failed to mature.

**Fig 5:**
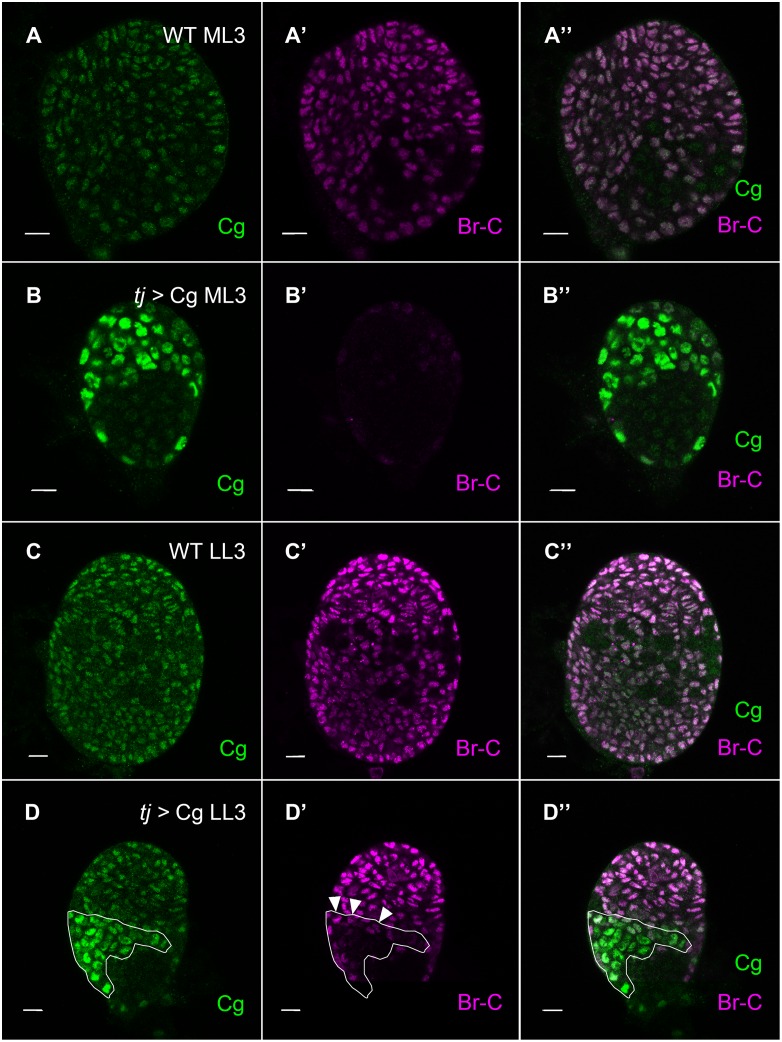
Over-expression of Cg abolishes Broad expression. In all panels, Anti-Cg is in green, Anti-BR-C is in magenta. (A, B) ML3 ovaries. In the wild type (A-A”) Cg and BR-C are both present in somatic nuclei in uniform levels. (B-B’) In Cg-over expressing ML3 ovaries, almost all somatic nuclei express high Cg levels and BR-C labeling is lost. (C-C”) Wild type LL3 ovaries, all somatic nuclei co-stain with BR-C and Cg. (D-D”) Regions of cells that over-express Cg (outlined) display reduced or no BR-C staining. Lack of BR-C expression is particularly apparent in the middle of the over-expressing region, where cells do not face wild type neighbors. Bars are 10 μm each.

The gross morphological abnormalities of Cg over-expressing ovaries resembled the developmental defects in ovaries over-expressing the dominant negative EcR [13]. Indeed, examination of Broad expression using an antibody directed against the core region of BR-C, which labels all Broad isoforms, revealed a strong Broad suppression in Cg over-expressing cells. At ML3, all somatic WT nuclei express BR-C (Fig 5A–5A’’). *tj*-Gal4, which is expressed in all somatic ovarian nuclei at this stage [5], led to Cg over-expression in most somatic nuclei (Fig 5B and 5B’’). Consequently, hardly any BR-C expression was observed in these nuclei (Fig 5B’ and 5B’’).

At LL3, due to the morphological defects caused by Cg over-expression, cells expressing high Cg levels were located at lateral ovarian regions (Fig 5D). In these cells, BR-C levels were very low or non-existent (Fig 5D’ and 5D’’). Interestingly, cells at the border of the region over-expressing Cg retained some BR-C labeling, suggesting a non-autonomous contribution to BR-C expression (Fig 5D’, arrowheads). Together, these results show that correct Broad expression requires wild-type levels of Cg, and any change in Cg amounts or function disrupts expression from this important locus.

### EcR binding to polytene chromosomes depends on correct Cg levels

**Fig 6:**
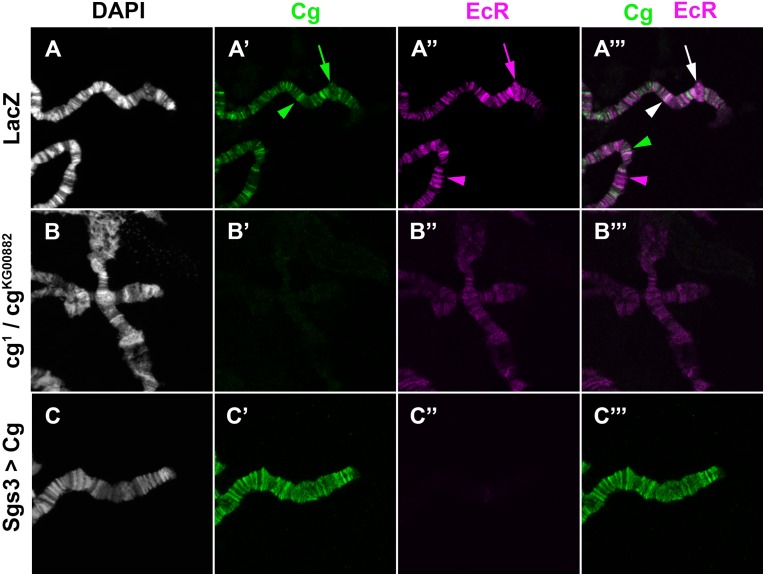
Cg affects EcR binding to polytene chromosomes. Salivary glands polytene chromosomes spreads. For all panels, imaging was performed with the same confocal settings. DAPI (white) stains DNA, anti-EcR is in magenta, and anti-Cg is in green. (A-A”) A portion of the X chromosome is shown. A green arrow in (A’) shows where Cg is bound to the *BR-C* puff. EcR is bound at the same puff (magenta arrow in A”). In the composite (A”‘), co-localization of the two antibodies appears in white. Two such bands are marked (white arrows). In addition numerous bands demonstrate unique EcR or Cg binding (magenta and green arrows, respectively). (B-B”‘) Chromosomes spread from *cg*^*1*^*/cg*^*KG00882*^ mutant larva. Chromatin bands are not as tight, very little Cg is observed and EcR binding to chromatin is reduced. (C-C”‘) Cg was over-expressed in salivary glands using *sgs3*-Gal4. A large amount of Cg, which spreads throughout the chromatin is observed. Little EcR can be detected on such chromosomes.

To determine how direct the control of Cg on *BR-C* expression is, we asked whether Cg is localized to the *BR-C* locus. At LL3, increased transcription from *BR-C* can be directly observed as puffing of the locus in polytene chromosomes of salivary glands [34]. Staining with anti-Cg revealed that Cg was localized to the *BR-C* puff in 84.5% of all chromosomes tested (n = 58, Fig 6A’ and 6A’’’, green arrow). Moreover, co-labeling with anti-EcR revealed that in all these cases, Cg co-localized with EcR (Fig 6A” and 6A’’’, white arrow). This suggests that transcription from *BR-C* may depend directly on Cg. EcR and Cg co-localized in some loci but not in others (Fig 6A’–6A’’’, green, magenta and white arrowheads). This suggests that Cg may not be an obligatory component of the EcR transcriptional complex. Alternatively, Cg may be present in some EcR-containing polytene chromosome bands at levels that are undetected by antibody staining, or associate with EcR complexes in a temporally-restricted manner.

As EcR and Cg affect BR-C expression, and both co-localized at the *BR-C* locus, we investigated whether EcR localization may depend on Cg. In the strong *cg*^*1*^*/cg*^*KG00882*^ allelic combination, lower Cg levels were detected on polytene chromosomes (100% of spreads, n = 18, Fig 6B’ and 6B’’’). On such chromosomes, EcR was still present, but at reduced levels (89% of spreads, n = 18, Fig 6B’’ and 6B’’’). In addition, the chromatin-banding pattern was loose, suggesting a global effect on chromatin by Cg (Fig 6B). Other allelic combinations displayed similar, albeit weaker phenotypes: The reduction in Cg levels in the weaker allelic combinations was not as severe, resulting in a smaller reduction in EcR binding to chromatin (S5 Fig).

Our data show that in the somatic cells of the ovary, a reduction in Cg protein results in lower Br-C induction, while over-expressing Cg results in an almost complete block of Br-C expression (Figs 3 and 5). We therefore investigated the status of EcR binding to polytene chromosomes of salivary glands that over-expressed Cg. In 45% of chromosome spreads, Cg was indeed over-expressed, coating the entire chromatin (n = 58, Fig 6C’ and 6C’’’). In such spreads, EcR binding was almost completely undetected (92% of Cg over-expressing spreads, Fig 6C’’ and 6C’’’). The effects of Cg levels on EcR binding to chromatin (reduction in the mutant background and severe reduction upon Cg over-expression), match the manner by which changes in Cg levels affect BR-C expression. Together, these results confirm that normal EcR activity depends on tight regulation over Cg levels and function.

### Cg promotes long-range chromatin contacts at the *BR-C* locus

The changes in chromatin structure observed in polytene chromosome of *cg*^*1*^*/cg*^*KG00882*^ animals, suggested that Cg may affect the chromatin structure at the *BR-C* locus. To test this possibility we used circularized chromosome conformation capture with high-throughput sequencing (4C). To compare chromatin conformation prior to- and following exposure to the ligand, and to ask how may Cg affect this response, we used Kc167 cells, which respond well to the ligand (see below). Since our observations in polytene chromosomes suggested a connection between EcR and Cg, we selected as viewpoints into the *BR-C* locus two regions that are associated with EcR. The first viewpoint, B1 (at 1609904), was located between the distal and proximal promoters, 2 Kilo-Bases (KB) away from EcR binding sites. The second viewpoint (B2, at 1619647) was close to the proximal promoter, in a region within the first intron, which binds EcR *in-vivo* (S6 Fig) [35,36,37].

**Fig 7:**
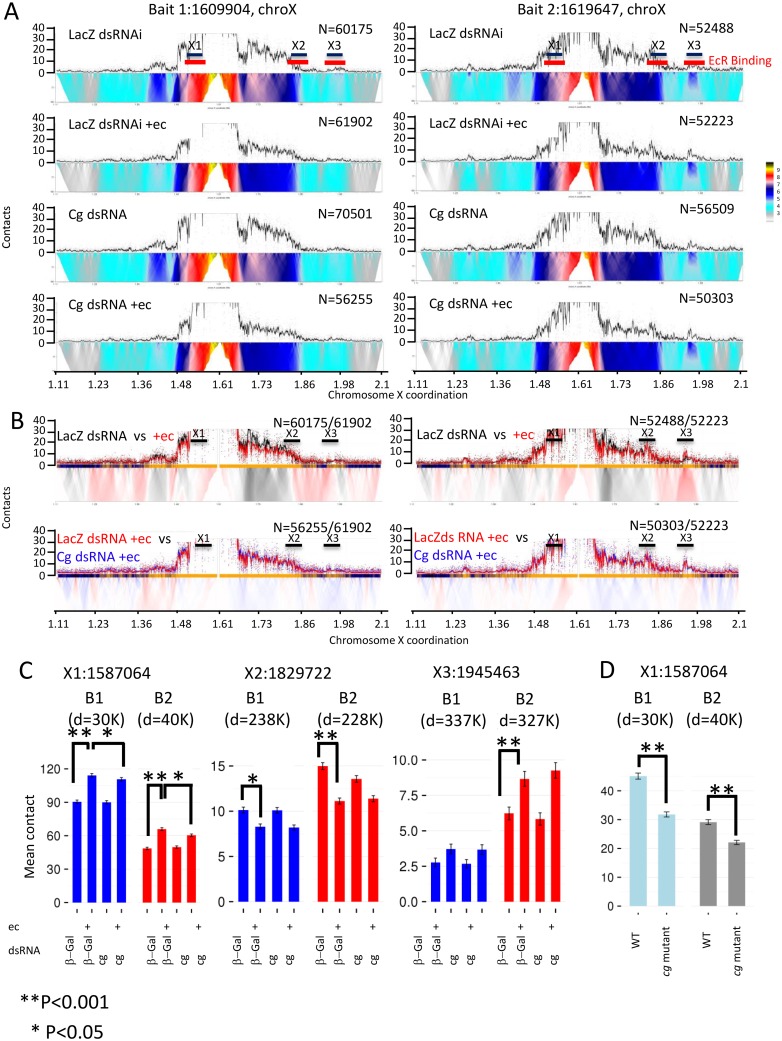
4C-seq profiles for *BR-C* locus-specific contacts in Kc167 cells and in wing imaginal discs. Regions within X chromosome are according to Ensembl_6 of GBrowse. (A) 4C domainogram for two different viewpoints (chromosome X, bait B1 1609904 on left, and bait B2 1619647 on right) and five experimental conditions are shown. The average contact intensity (smoothed number of captured ligations) is depicted as a trend with error band representing two standard deviation of the estimated mean. Raw data points are shown as small black dots. A color-coded depiction of contact intensity in increasing scales (10 to 300 restriction fragments) is shown below each trend. X1-3 indicates the position of loci we select for quantitative analysis below. N, represent the number of inferred ligation evens used to generate each profile, typically based on 1-3M sequenced reads. (B) Comparison of 4C profiles in control cells treated with ecdysone (red) to untreated cells (black, top) or treated cells in which Cg was knocked down (blue, bottom). Each comparison shows overlaid contact intensity trends on top. The domainogram is showing the differential contact intensity in increasing genomic window sizes. Red bands on the dominogram indicates contacts in the region are stronger following ecdysone treatment. (C) Bar-plots represent the mean contacts for windows X1, X2, 200 fragments per each and 30 fragments for X3. The number of contacts per window is color-coded. (D) Bar-plots representing the mean contacts for X1 with viewpoints B1 and B2 in wing imaginal discs derived from wild type and *cg*^*1*^*/cg*^*KG00882*^ animals, 40 fragments. **P<0.001, *P<0.05 (Chi-Square pair-wise test).

We observed a domain-like contact landscape around the B1 viewpoint, with asymmetric contacts extending some ~250KB downstream (Fig 7A, color coded domainogram), and ~125KB upstream. Analysis of B2 indicated several long-range contacts that were enriched over the typical domain architecture (Fig 7A, right). We selected the three loci with the strongest evidence for long-range contacts for further quantitative analysis (X1-X3). Significantly, these three contact points constituted the three sites with the highest EcR-binding content within this ~370KB region (marked as red bars underneath X1-X3, Fig 7A, right, S6 Fig) [36,37]. Thus, the EcR binding region within *BR-C* is engaged in long-range contacts with other EcR-binding regions.

We compared systematically the 4C contact profiles around the B1 and B2 viewpoints between conditions, using differential contact analysis at multiple scales (Fig 7B). Quantitative follow up of the contact intensity at the X1, X2 and X3 loci (Fig 7C) showed that the overall contact architecture was stable. However, ligand exposure and Cg presence modified some contacts in significant ways: The closest contact (X1) was located ~30KBs upstream, next to the distal *BR-C* promoter. Our data indicated about 27%-22% increase in the number of contacts between X1 and either B1 or B2, respectively, following ecdysone treatment (Fig 7C). Notably, a significantly weaker increase was detected when cells were pre-treated with *cg* ds-RNA. Thus, exposure to ecdysone increases *BR-C* transcription and contact stability. Both these phenomena are Cg-dependent. Reciprocal 4C confirmed the increased number of contacts between B1 and B2 to X1 upon ecdysone treatment and strengthened the dependency of this interaction on Cg (S7 Fig).

To further substantiate the effects of Cg-depletion on the *BR-C* locus, we studied its conformation in wing imaginal discs derived from wild type and from *cg*^*1*^*/cg*^*KG00882*^ late third instar animals. At this developmental stage, ecdyosne titers are high, and *BR-C* transcription peaks. Contacts between X1 and B1 or B2 were significantly lower in *cg*-mutant animals as compared to wild type (Fig 7D). This conforms to the reduced BR-C expression and reduced EcR- binding to the locus that is observed in mutant animals. Thus, in-vivo studies confirm the dependency of contacts at the BR-C locus on normal Cg levels.

The second contact region (X2) was ~228KB downstream of B2, within *CG42666*, and showed an ecdysone -dependent decrease in contacts with B1 and B2 (Fig 7C). Finally, The most distant contact (X3) was ~327KB downstream of B2, within the first intron of *DHR4*. X3 showed a significant increase in contacts with B2 upon ecdysone treatment. Contacts between B1/B2 and X2 or X3 were not affected by *cg* knockdown (Fig 7C).

In summary, our 4C analysis showed a complex chromatin structure at the *BR-C* locus, featuring long-range contacts between EcR-binding regions across ~370KB. These connections are differentially modulated by exposure to ligand, but only the contacts within the *BR-C* locus are Cg-dependent. 4C profiles around *BR-C* serve as a preliminary indication to the diversity of changes both ligand and Cg confer upon the *BR-C* chromatin landscape.

### Cg is required for proper EcR response in Kc167 cells

Our results demonstrate that EcR binding regions in *BR-C* are associated with two other genes containing EcR-binding regions, and that EcR binding to chromatin depends on Cg. This prompted us to ask whether Cg and EcR might physically interact, and whether Cg may also affect the transcription of DHR4 and CG24666. Our attempts to co-immunoprecipitate EcR and Cg from either adult ovaries or Kc167 cells were unsuccessful. Thus, either the two proteins do not physically interact, or they are part of a temporally restricted or labile complex. To examine this last option, we measured EcR-Cg interactions using a proximity ligation assay (PLA), which can detect physically associated proteins without cell disruption.

**Fig 8:**
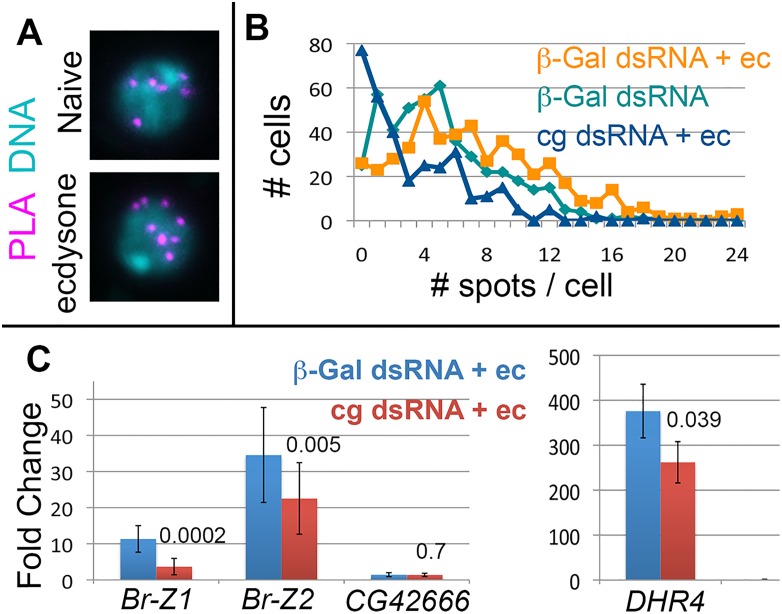
Cg is required for EcR-mediated transcription. (A) Representative data from a PLA experiment. A compression of a few Z sections is shown. PLA signal is in magenta and DNA (DAPI) is in blue. Complexes appear in control, untreated β-Gal dsRNA cells (naïve), and their numbers increase following treatment with ecdysone. (B) Summary of PLA data for control β-Gal dsRNA cells (teal), β-Gal dsRNA cells following ecdysone treatment (orange) and *cg* dsRNA following ecdysone treatment (Blue). The plot shows the number of cells (Y axis) containing a particular number of PLA spots (from 0 to 24, X axis). (C) qPCR quantification of fold-increase in *BR-Z1*, *BR-Z2*, *CG42666* and *DHR4*, following ecdysone treatment, as compared to transcripts levels in cells that were not treated by ecdysone. Control, *β-Gal*-dsRNA is in blue and *cg*-dsRNA in red. Paired t-test p values are indicated.

Control, β-Gal ds-RNA, cells contained an average of 5.09 PLA spots located at their nuclei (n = 461 cells, Fig 8A and 8B). Treatment of control cells with 1μM ecdysone for 2 hours increased the average number of spots to 7.26 (n = 490 cells, Fig 8A and 8B). A Mann-Whitney U test determined that this change is highly significant (p vaue 3.54E-13). *cg* dsRNA cells treated with ecdysone contained only 3.27 spots on average (n = 320, Fig 8B, p value 1.06E-35, compared to ecdysone treated control cells), attesting to the specificity of the assay. Together, the PLA and polytene chromosome studies suggests that only a fraction of Cg and EcR may reside within the same complex. Some of these complexes may be preformed, while others either form de-novo, or are stabilized upon exposure to ecdysone.

We next tested how may Cg affect transcription of genes in contact with *BR-C*. We first measured isoform-specific induction of *BR-C* mRNA levels upon ecdysone treatment. Control Kc167 cells treated with dsRNA against β-Gal, responded to ecdysone exposure by up-regulating both *BR-Z1* and *BR-Z2* transcripts, with *BR-Z2* induction consistently higher than *BR-Z1* (Fig 8C). When *cg* levels were reduced by *cg*-dsRNA (S8 Fig), up-regulation of either transcript was attenuated by about half (Fig 8C). The dependence of *BR-C* expression on Cg in both gonadal and Kc167 cells, which originate from embryonic blood cells, suggests that control of this locus by Cg is a ubiquitous phenomenon.

The first associated gene, CG42666, lying next to X2, was not induced by ecdysone (Fig 8C). Since contacts between BR-C and CG42666 were reduced by ligand treatment (Fig 7C), this suggests that following exposure to ligand, some long-range contacts with inactive EcR targets may be diminishing.

On the other hand, transcription of DHR4 (which encompasses X3) was greatly increased by ecdysone treatment. Induction of this gene was an order of magnitude higher than that of *BR-C* (Fig 8C). Contacts between BR-C and DHR4 increased upon ligand exposure (Fig 7). Thus, the contacts with the un-induced gene diminish, while those with the induced gene increase. Significantly, DHR4 transcriptional induction by ecdysone was reduced in *cg*-RNAi cells. Thus, Cg can affect other ecdysone-induced genes besides *BR-C*.

## Discussion

The *BR-C* locus controls a variety of processes ranging from stem cell establishment, cell death, neural network organization to animal behavior [13,18,19,20,21,22,23]. Despite its central role in fly biology, little is known about how this complex locus is being controlled. Our work shows that correct levels and function of the chromatin binding protein Combgap are required for Br-C expression. We further show that EcR-enriched regions within *BR-C* are engaged in long-range associations with each-other and with other EcR-enriched loci. Lastly, our data indicate Cg affects transcription of EcR-induced genes by controlling the access of EcR to chromatin.

### Cg and EcR-mediated *BR-C* expression

Cg is associated with the *BR-C* locus and its RNA and protein products in three different cellular systems: somatic ovarian cells, Kc167 cells and in salivary glands. One major inducer of transcription from this locus is EcR [15,17,34]. Several lines of evidence suggest that Cg effects *BR-C* via EcR. First, EcR and Cg co-localize at the *BR-C* locus. Second, we observe reduced EcR binding to polytene chromosomes in *cg* mutants. Third, EcR binding to chromatin is almost completely abolished when Cg is over-expressed in salivary glands. These changes in EcR binding to chromatin fit very well with how Cg levels affect BR-C in somatic ovarian cells. We observe a reduction in Br-Z2 levels in *cg*-mutant clones, and a complete block of all BR-C isoforms in cells over-expressing Cg. Cg levels affect EcR in a global manner, suggesting other EcR targets are likely affected. Indeed, the transcription of another EcR target, DHR4, is also reduced in *cg*-RNAi Kc167 cells.

Lastly, the PLA approach suggests that at least some nuclear complexes contain both EcR and Cg. It is likely that EcR and Cg do not directly bind each other, as co-IP approaches failed to establish a direct connection. In addition, Cg is not dedicated exclusively to the ecdysone pathway, and also affects Wingless and Hedgehog target genes [2,3,4]. Indeed, the amount of PLA spots and co-stained bands in salivary glands suggest that only a minor fraction of Cg is engaged in such stable complexes. We hypothesize Cg may have pleiotropic roles, and engage in different or more general complexes. Indeed, a recent publication identified Cg as a PRE-binding protein, and as co-localizing with pleiohomeotic and polyhomeotic (Ph) [38]. It is unclear if this interaction overlaps with how Cg affects EcR and the *BR-C* locus, or if it represents an entirely independent function. The exact biochemical function of Cg as a more general mediator of protein binding onto chromatin requires further investigation.

### Association between EcR-enriched loci

4C analysis shows that EcR-enriched regions within the first intron of *BR-C* contact EcR-enriched binding site close to the distal *BR-C* promoter, ~30KBs upstream. The interactions between these EcR-enriched regions existed in Kc167 cells prior to exposure to ecdysone, but increased following ligand binding. Our results support a model where the chromatin is pre-programmed for specific transcriptional responses, and ligand exposure can modulate the intensity of these pre-formed connections. The ecdysone response is rapid, and would benefit from a pre-arranged chromatin structure that enables a coordinated transcriptional response for the hormone.

The contacts of EcR-enriched regions between *BR-C*, *CG42666* and *DHR4* suggest that BR-C is transcribed in association with other genes, or in a ‘multigene’ complex. Such complexes are prevalent in mammalian genomes [39]. Interestingly, contact intensity increases upon ecdysone treatment for *BR-C* and *DHR4*, the two genes displaying ecdysone-induced transcription. Contacts intensity is reduced for *CG42666*, which does not respond to ecdysone treatment. At this stage we cannot determine whether the changes in chromosome conformation participate in regulating ecdysone-induced transcription.

Within the *Br-C* locus, increased contact intensity is Cg-dependent. The decreased intensity following *cg*-reduction is observed in both Kc167 cells, and to a larger extent, in *cg*-mutant animals. The consistency of the effect in cells of different origin suggests that Cg affects the conformation of DNA at the BR-C locus in a global manner. The larger effect in the mutant animals is likely due to the inefficiency of the dsRNA technique in tissue culture.

Unlike the contacts between the distal *BR-C* promoter and the *BR-C* first intron, the contacts with *DHR4* are Cg-independent. As Cg affects the transcription of both *BR-C* and *DHR4*, this adds to the complexity of the regulatory interactions in this region. In a similar vein, qPCR measurements show an order of magnitude difference in ecdysone induction levels between *BR-C* and *DHR4*. Thus, induction strength is not determined in this case by the association *per-se*, but by individual promoters and/or the existence of other elements such as insulators, which participate in ecdysone-induced chromatin looping [35,40]. Whether Cg may participate in these complexes or even mediate some or their differences await biochemical analysis.

### Cg, *BR-C* transcription, and gonad morphogenesis

While qPCR measurements in Kc167 cells show *cg*-dsRNA reduces transcription of both *BR-Z2* and *BR-Z1*, protein measurements in somatic ovarian clones show a marked reduction only for BR-Z2 protein levels. One option to explain this difference involves the smaller reduction of *BR-Z1* transcripts in *cg*-dsRNA as compared to *BR-Z2*. This smaller difference may still be detected by qPCR, but not by antibodies. A second, not mutually exclusive explanation may be connected to post-transcriptional control. The reduced *BR-Z1* levels may trigger some compensation at the level of splicing or protein translation. Alternatively, the manner by which Cg controls transcription from the *BR-C* locus in ovarian and Kc167 cells may be somewhat different.

BR-Z1 protein levels are reduced in whole-body mutants but not in clones, suggesting a systemic component for *cg*-depletion on ovarian *BR-C* transcription. During late larval stages, BR-C acts in a feed-forward loop, augmenting ecdysone production [41]. Cg may therefore affect ovarian *BR-C* expression both autonomously and systemically. An antagonistic effect may be caused by DHR4, which is a negative regulator of ecdysone production [42] and is also reduced by *cg*-depletion. How these two opposing effects balance out in the prothoracic gland, which synthesizes ecdysone, remains to be investigated. However, *cg*-mutant larvae, which do not pupariate, suggest that Cg depletion results in insufficient ecdysone production.

Finally, our previous work showed that ecdysone signaling promotes three processes during gonadal development: gonadal growth, niche differentiation, and PGC differentiation. ecdysone signaling in the larval ovary promotes BR-Z1 expression exactly at the time when niches and PGCs differentiate. This suggests that BR-Z1 is necessary for stem cell unit differentiation. Indeed, expression of a dominant negative EcR abolishes both BR-Z1 expression and stem cell unit differentiation [13]. BR-Z2 is expressed even prior to stem cell unit differentiation, and therefore cannot be sufficient to induce differentiation. However, BR-Z2 may still be necessary for the correct differentiation of niches and PGCs, together with BR-Z1. Indeed, BR-Z2 could partially rescue both ovarian growth and niche differentiation.

One important difference between the effects of BR-C and Cg on gonad morphogenesis is that while both factors promote ovarian growth and niche formation, Cg retards PGC differentiation while BR-C promotes it. This suggests that Cg affects other targets important for gonadal growth. Previous work uncovered many signaling pathways that are important for niche formation and GSC establishment [7]. Understanding how these pathways converge to form the organized organ is crucial for basic understanding of organogenesis and for applications in regeneration. Uncovering how pleiotropic proteins such as Cg contribute to coordinated transcription and hence to organogenesis will be part of this effort.

## Materials and Methods

### Fly stocks

*cg*^*KG00882*^, *cg*^*1*^, *FRT42D*, *FRT42D*,*ubi*-GFP, Cg-GFP (*cg*^*CC01469*^), *cg-RNAi* (*cg*^*HMS01145*^), UAS-Br-Z1, UAS-Br-Z3, UAS-Br-Z4 were from the Bloomington Stock collection. *cg*^*2*^ was from Dr. William J. Brook (University of Calgary, Canada), *tj*-Gal4, *nos*-Gal4 from Dr. Ruth Lehmann (NYU, USA), *c587*-Gal4 was from Dr. Ting Xie (Stwoers Institute, USA), UAS-Cg (P{y+, Mae-UAS6.11}Cg was from Dr. John Merriam (UCLA, USA). UAS-Br-Z2 was prepared as follows: br-Z2 cDNA in pBS(SK-)was obtained from Dr. Lauren von Kalm (UCF, USA). Br-Z2 was PCR-amplified using the forward primer AAAAGAATTCAGCCAGAACCAGACACCCATCGAGATG and the reverse primer TTTTAGATCTCATGGTCGTGCTGTCTTTCATCGCTG. The PCR product was suBR-Cloned into pUAStattB via the BglII and EcoRI sites. Injection of UAS-Br-Z2 into the attP2 site on the third chromosome were performed by Genetic Services (Sudbury, MA, USA).

### Larval dissections and staining

Larval developmental timing was determined as described previously [13]. In short, crosses were performed in bottles, to ensure a sparse larval population. In these strict under-crowded conditions, larval development is uniform. Laying was performed for two hours, and eggs were allowed to mature at 25°C until late third instar (120 h after laying). *cg* mutants, which do not pupariate, were dissected 5–7 days after laying. Females were dissected and stained as previously described [13,43].

### Antibodies

Generation of BR-Z2-specific antibodies was according to [44]: The Br-Z2 isoform-specific region was PCR amplified (forward primer: GGATCCAAGGAAACTCGCCCAAGAAACTC, reverse primer: GAATTCTGCTGTGGCTGTTGGCTTTGC). The amplified region was cloned using BamHI and EcoRI, into pGEX-3X bacterial expression vector in E. coli BL21 strain. Bacterial cultures were grown until OD_600nm_ 0.5 and induced with 2mM IPTG for 4h. GST fusion proteins were purified on glutathione agarose (Sigma). The purified GST-BrZ2 was used to immunize New Zealand female rabbits. The first injection was in Freund's complete adjuvant and the boost, spaced for at least 3 weeks, was in Freund's incomplete adjuvant. A total of 3 boosts were performed and sera taken 2 weeks after the last boost was tested for immunoreactivity against the injected proteins. The resulting Ab reacts specifically with Br-Z2 (S9 and S10 Figs).

Anti-Cg (1:1000) was a kind gift from Dr. William J. Brook (University of Calgary, Canada), Rabbit-anti-GFP (1:1000) was from Invitrogen, Rabbit-anti-Vasa (1:5000) was a gift from Dr. Ruth Lehmann (NYU, USA), Guinea Pig anti-Tj (1:7000) was a gift from Dr. Dorothea Godt (Univeristy of Toronto, Canada). The following monoclonal antibodies were obtained from the Developmental Studies Hybridoma Bank, developed under the auspices of the NICHD and maintained by the University of Iowa, Department of Biology: anti-En (4D9, 1:20), anti-Hts (1B1, 1:20), anti-BR-C (25E9.D7, 1:10), anti-Br-Z1 (Z1.3C11.OA1, 1:10), anti-Orb (6H4, 1:20), anti-EcR.C (AG10.2, 1:20). Larval ovaries staging and staining was as previously published [13].

### qPCR

0.4x10^6^ cells were seeded together with the dsRNA and RNA isolated 3 days later using PerfectPure RNA cultured cell kit (5Prime) according to manufacturer’s instructions. Reverse transcription was performed with the High Capacity cDNA Reverse Transcription Kit (Applied Biosystems). qPCR employed SYBR Green (Invitrogen) with the following primers (forward and Reverse) RpS17: CAAGATTGCCGGCTATGTCA and CCTGCAACTTGATGGAGATACCA. Combgap: TCCCCGAAGACCGAACTACA and GTACGGGCGTTCCTTCTTGA, CG42666: GGGCTAGGGACGACAGTTT and GTTTGGAAGCTCGCTACTGG, DHR4: CGCTCCTACCTGCAAAACTC and CACGAAGGGCACATAGAACA, or the Taqman assay: RpL32 (Dm02151827), Br-Z1 (Dm01837161_m1), Br-Z2 (Dm01821011_m1). Q-PCR was performed in Applied Biosystems’ StepOne TM, analyzed by DDCT and normalized to RpS17 or RPL32. For statistical analyses, two-tailed student’s T-test were performed. P values are indicated.

### Tissue culture

Kc167 cells were maintained at 25°C in *Shields and Sang* M3 insect medium (Sigma) containing 10% fetal bovine serum (Gibco) and 1% penicillin–streptomycin (Gibco). For RNAi, dsRNA synthesis was according to DRSC protocol using Readymix (Sigma), MEGAscript T7 kit (Ambion) and RNAeasy (Qiagen). Amplicons from the DRSC database DRSC32301 for *combgap* and DRSC24562 for *β-Gal* were used. 2 μg of dsRNA against either *combgap* or *LacZ* together with 2 μl DharmaFECT4 (Dharmacon) were applied to 0.4x10^6^ cells in 12-well plates. Cells were exposed to dsRNA for 72 hours and then 1μM ecdysone or control 95% Ethanol were added to the medium for 2 hours. Following ecdysone exposure, cells were harvested for qPCR.

### Proximity ligation assay (PLA)

PLA was performed according to manufacturer’s specifications using the Duolink kit (Sigma-Aldrich) with the PLA probe anti-Rabbit PLUS (DUO92002), the anti mouse Minus (DUO92004) and the detection reagent red (DUO92008). First antibodies used were Rabbit-anti-Cg (1:1000) and mouse-anti-EcR (1:20).

### 4C-Seq

The preparation of 4C template (five biological repeats) was performed as described previously [45], with some modifications. 5X10^6^ Kc167 cells were fixed and following cross-linking, the DNA was digested by the 4 cutter restriction enzyme DpnII (NEB), ligated by high concentrated T4 ligase (NEB). The ligation products were sonicated in to fragments at the average length of 500 bp, end repaired by fill-in and exonuclease reaction and then ligated to an adaptor sequence for the Illumina Trueseq process. 150–200 ng of the resulting 4C template were used for 2 sequential PCR reactions. The first PCR was performed by primers designed to target the region of our interest and illumina Truseq enrichment PCR primer 2. In the second PCR, we used primers that include the sequence illumina Truseq universal adapter AATGATACGGCGACCACCGAGATCTACACTCTTTCCCTACACGACGCTCTTCCGATCT followed by specific internal sequence together with illumina Truseq enrichment PCR primer 2 (see below). The primers for the first PCR were designed to target region that is far by 80 to 98 bp from the DpnII site. The primers (internal primes) for the second PCR were designed to target the region that is far by around 3–80 bp from the same DpnII site.

The primers that were used for two BR 4C viewpoints (B1, B2) and for the primers that were used for three contact regions (X1, X2, X3) are listed in the following table 2.

**Table 2:** Primers used for 4C analysis.

	ChroX coordinate	Forward primer	Internal sequence of the nested forward primer
B1	1609904	TCGTGCACTTGACTATCTTCGTG	TCGTGCACTTGACTATCTTCGTG
B2	1619649	GAAGCGAGACAAAAGTGGTGAGG	ATAGTTCCAGACAGGAAAGAGGGG
X1	1587064	TCAAAGTCGGGCACTATATGTTGTGTG	CAACGCCCGATTTCAACAAAGATTC
X2	1829722	CTTGGCCAATTACTATGTGAAATCCCG	GTACATGCCTCCCTTCCCCCGCACTT
X3	1945463	TTGTCACAGCTATCAAATGGCAAGAGG	TCCTTTCAACTTAGCCCGAGATTCGAT

Sequenced products were mapped to the genome using an in house pipeline (available upon request), and the precise sonication sites were used to eliminate PCR duplicates. 4C domainograms [46] show a smoothed contact intensity profile with gray error band representing 2 SD on the estimated mean contact intensity in a running window. It also shows a color-coded visualization of contact intensities in a series of increasing scales (from 10 restriction fragments to 300 restriction fragments). As the modified protocol provide precise control over PCR duplicates, linear means of the number of ligation evens per restriction fragment within windows can be used. Similarly, statistical tests comparing the total number of ligations in a given window between two conditions (following normalization for total sequence coverage) are robust. This was reconfirmed for the current experiments by quantifying technical variance on triplicate experiments.

### Imaginal Discs harvesting for 4C analysis

Inverted head segments of 400 OR (wild type) or 200 *cg*^*KG000882*^/*cg*^*1*^ animals were fixed in 2% formaldehyde in PBS (20 min., Room Temperature). Fixed tissue was then washed twice with PBS/125mM Glycine/0.01% Triton-X-100, and a third time with PBS. Samples were washed again with PBS/1% protease inhibitor cocktail (P8340, Sigma). Wing Imaginal discs were then dissected out and placed in an eppendorf tube. Excess fluid was removed and tissues were snap-freezed in Liquid Nitrogen and stored at -80°C. To prepare DNA, discs were thawed on ice and centrifuged at 13,000 rpm for 20 sec. Excess fluid was discarded. Discs were resuspended in 50 ul lysis buffer (10 mM Tris-HCl pH 8.0, 10 mM NaCl, 0.2% Igepal CA360 (Sigma I8896)), and 10 ul/ml of protease inhibitors (Sigma P8340). Discs were homogenized with a plastic motorized pestle (3X2min). Following a brief centrifugation, 500 ul of lysis buffer and 50 ul of protease inhibitors were added and the suspension was centrifuges at 5000 rpm for 5 min, at RT. Lysate was washed twice with ice-cold 1.2X NEBuffer 3 at 5000 rpm, 5 min, at RT, and resuspended in 500 ul NEBuffer 3, supplemented with 7.5 ul 20% SDS. Mixture was incubated at 37°C, rotating at 900 rpm for 1h in a Thermomixer, followed by an addition of 50 ul of 20% T-X-100 and an additional incubation at 37°C, rotating at 900 rpm for 1h in a Thermomixer. Lysate was digested with 400 U of DpnII at 37°C, 900 rpm overnight in a Thermomixer. Enzyme inactivated at 65°C, 20 min. Ligation and continuation of the 4C protocol was as performed for KC cells.

### Polytene chromosome squash

Larvae were grown at 18°C in a non-crowded culture. Wandering 3^rd^ instar larvae were washed and dissected in Ringer’s buffer. Extracted salivary glands were spread according to published protocol [47]. Rabbit-anti-Cg (1:1000) and mouse-anti-EcR (1:20) were used to localize EcR and Cg to the *broad* locus, and DAPI staining was used to highlight the banding pattern. The *br* locus was identified by the banding pattern that is specific to the tip of the X chromosome and by the puffing pattern specific to the ecdysone response at late third instar.

## Supporting Information

S1 Fig Cg is required in somatic cells. In all panels, anti-Cg is in green. (A) Anti-Hts (magenta) outlines somatic cells and fusomes within PGCs. The germline driver *nos*-Gal4 removes Cg specifically from PGCs (outlined in A, A’). Fusomes in PGCs remain spherical (inset) showing germ cells have not differentiated into cysts. (B, B’) Control LL3 ovaries showing Cg in all nuclei and well-formed TFs (anti-En, magenta). (C, C’) the somatic driver *tj*-Gal4 drives both DicerII and an RNAi construct directed against Cg. Cg is still apparent in germ cell nuclei and in anterior nuclei where tj-Gal4 is not expressed. However, remnants of Cg protein can still be seen in nuclei throughout the ovary. (D, D’) tj-Gal4 drives DicerII and GFP^RNAi^ in Cg-GFP ovaries. Cg is still expressed in PGCs, while very little Cg can be observed in somatic nuclei. PGCs do not differentiate and carry spherical fusomes (D’, arrowheads, anti-Hts, magenta).
(TIF)

S2 Fig Cg mutants become giant larvae. *cg*^*1*^ or *cg*^*KG00882*^ alleles were balanced on the attached chromosome SM6a-TM6,Tb, such that homozygous larvae were easily recognized by their lack of Tb phenotype. A control LacZ larva at the wandering stage, 5 days after egg laying is shown for comparison. *cg*^*1*^ or *cg*^*KG00882*^ giant larvae were collected from a bottle 8 days after egg laying. The mutants were still at the larval stage, while their heterozygote siblings were already pupae.
(TIF)

S3 Fig Br-Z1 is not reduced in *cg*-mutant clones. In all panels, Anti-Br-Z1 is in magenta and anti-GFP is in green. Mutant cells lack GFP and are outlined. Similar levels of Br-Z1 protein are present in *cg*^*KG00882*^ (A, A’) or *cg*^*2*^ (B, B’) mutant clones as compared with their WT neighbors. Bar is 10 μm for all panels.
(TIF)

S4 Fig Partial rescue of gonad morphogenesis by Br-Z2 over-expression. Anti-Br-Z2 is in green or grey. Anti-Hts outlines somatic cells and labels fusomes within PGCs (magenta in A, B, C, D). Anti-En (magenta in A”, B”, C”, D”) labels TFs. (A, A’, A”) control ovaries, showing normal Br-Z2 expression and normal TFs. (B, B’, B”) Over-expression of Br-Z2 in a WT background, showing that over-expressing this protein does not result in a large BR-Z2 increase above WT levels and does not change normal ovarian development. (C, C’, C”) *cg*-mutant ovaries showing reduced size, severe defects in TF formation, no posterior somatic cells, and very little expression of Br-Z2. (D, D’, D”) *cg*-mutant ovaries over-expressing Br-Z2, ovaries increase in size compared to *cg*-mutants (compare to S4C), contain more TFs (compare to S4C”, Table 1), and have a sizable population of posterior somatic cells. However, rescued ovaries do not reach the advanced developmental stage of WT ovaries (compare to S4A).
(TIF)

S5 Fig Reduced Cg and EcR binding to polytene chromosomes in *cg* mutants. Spreads of polytene chromosomes from salivary glands were stained with Dapi (white), anti-Cg (green) and anti-EcR (magenta). Glands were stained and imaged using the same confocal settings and on the same day as those of WT (Compare to Fig 6). Cg staining is weaker than WT in the *cg*^*1*^*/cg*^*2*^ (A-A”‘) and *cg*^*2*^*/cg*^*KG00882*^ (B-B”‘). The weakening in EcR staining correlates with the level of Cg protein remaining on the polytene chromosomes.
(TIF)

S6 Fig Location of viewpoints and X1-X3 in relation to the *BR-C* locus. Gene structure and genomic positions are according to release 6. The locations of the EcR-enriched binding regions are in red, and the viewpoints are in blue.
(TIF)

S7 Fig Reciprocal 4C for X1-X3 with B1/B2 within the *BR-C* locus. Bar plots showing the mean contact of the viewpoints (X1, X2, X3) with B1 and B2. For windows B1, B2, interaction with X1 30 fragments per each and 200 fragments for X3 interaction with B1, B2. While the number of contacts between B1/B2 and X2/X3 is low, the tendencies of interactions remain similar in the reciprocal 4C. *P<0.001 (Chi-Square pair-wise test).
(TIF)

S8 Fig Reduction of *cg* expression in Kc167 cells. Cells were treated with either control, β-Gal ds-RNA or with *cg* ds-RNA. *cg* mRNA levels were measured by qPCR. The data presented is derived from 5 biological repeats.
(TIF)

S9 Fig Specificity of anti-Br-Z2 antibodies. Ovaries were stained with Anti-Br-Z2 (green or white) and with anti-Hts (magenta). PGCs are outlined. Control ovaries (nos-Gal4>b-Gal) show anti-Z2 expression only in somatic cells, but not in PGCs. Each of the four BR-Z isoforms was expressed in germ cells using the driver nos-Gal4. The BR-Z2 antibody stains germ cells only upon expression of BR-Z2, attesting to the specificity of the antibody.
(TIF)

S10 Fig Specificity of anti-Br-Z2 antibodies. Western Blot analysis of imaginal discs using various anti-Br antibodies. Anti-BR-C recognizes the two major BR-C isoforms that are expressed in the discs (BR-Z1 and BR-Z2, indicated). Anti-BR-Z2 and anti-BR-Z1 each recognizes an individual isoform.
(TIF)

S1 Video Third instar wild type gonads of the genotype dicer2; *t**j*-Gal4, Cg-GFP; UAS-β-Gal were dissected and stained with anti-En (magenta), which labels TFs. A complete Z section of a representative gonad is shown. TFs are regularly spaced and have all matured.
(AVI)

S2 Video Third instar gonads of the genotype dicer2; *t**j*-Gal4, Cg-GFP; UAS-GFP^RNAi^, in which *cg* is reduced, were dissected and stained with anti-En (magenta), which labels TFs. Gonads are smaller than WT (compare to S1 video). Less TFs are present than in WT ovaries. While some TFs are long and mature, others are shorter.
(AVI)

## References

[1] Lindsley DL, Zimm GG (1992) The Genome of Drosophila melanogaster. San Diego: Academic Press.

[2] Song Y, Chung S, Kunes S (2000) Combgap relays wingless signal reception to the determination of cortical cell fate in the Drosophila visual system. Mol Cell 6: 1143–1154. 10.1016/S1097-2765(00)00112-X 11106753

[3] Campbell GL, Tomlinson A (2000) Transcriptional regulation of the Hedgehog effector CI by the zinc-finger gene combgap. Development 127: 4095–4103. 10976042 10.1242/dev.127.19.4095

[4] Svendsen PC, Marshall SD, Kyba M, Brook WJ (2000) The combgap locus encodes a zinc-finger protein that regulates cubitus interruptus during limb development in Drosophila melanogaster. Development 127: 4083–4093. 10976041 10.1242/dev.127.19.4083

[5] Gilboa L, Lehmann R (2006) Soma-germline interactions coordinate homeostasis and growth in the Drosophila gonad. Nature 443: 97–100. 10.1038/nature05068 16936717

[6] Zhu CH, Xie T (2003) Clonal expansion of ovarian germline stem cells during niche formation in Drosophila. Development 130: 2579–2588. 10.1242/dev.00499 12736203

[7] Gilboa L (2015) Organizing stem cell units in the Drosophila ovary. Curr Opin Genet Dev 32C: 31–36. 10.1016/j.gde.2015.01.005 25703842

[8] Lengil T, Gancz D, Gilboa L (2015) Activin signaling balances proliferation and differentiation of ovarian niche precursors and enables adjustment of niche numbers. Development 142: 883–892. 10.1242/dev.113902 25633355

[9] Godt D, Laski FA (1995) Mechanisms of cell rearrangement and cell recruitment in Drosophila ovary morphogenesis and the requirement of bric a brac. Development 121: 173–187. 7867498 10.1242/dev.121.1.173

[10] Sahut-Barnola I, Dastugue B, Couderc J-L (1996) Terminal filament cell organization in the larval ovary of Drosophila melanogaster: ultrastructural observations and pattern of divisions. Roux's archives of developmental biology 205: 356–363. 10.1007/BF00377215 28306086

[11] Sahut-Barnola I, Godt D, Laski FA, Couderc JL (1995) Drosophila ovary morphogenesis: analysis of terminal filament formation and identification of a gene required for this process. Dev Biol 170: 127–135. 10.1006/dbio.1995.1201 7601303

[12] Green DA 2nd, Extavour CG (2012) Convergent evolution of a reproductive trait through distinct developmental mechanisms in Drosophila. Dev Biol 372: 120–130. 10.1016/j.ydbio.2012.09.014 23022298

[13] Gancz D, Lengil T, Gilboa L (2011) Coordinated regulation of niche and stem cell precursors by hormonal signaling. PLoS Biol 9: e1001202 10.1371/journal.pbio.1001202 22131903 PMC3222635

[14] Song X, Zhu CH, Doan C, Xie T (2002) Germline stem cells anchored by adherens junctions in the Drosophila ovary niches. Science 296: 1855–1857. 10.1126/science.1069871 12052957

[15] Chao AT, Guild GM (1986) Molecular analysis of the ecdysterone-inducible 2B5 "early' puff in Drosophila melanogaster. EMBO J 5: 143–150. 3007111 10.1002/j.1460-2075.1986.tb04188.x PMC1166706

[16] Bayer CA, Holley B, Fristrom JW (1996) A switch in broad-complex zinc-finger isoform expression is regulated posttranscriptionally during the metamorphosis of Drosophila imaginal discs. Dev Biol 177: 1–14. 10.1006/dbio.1996.0140 8660872

[17] DiBello PR, Withers DA, Bayer CA, Fristrom JW, Guild GM (1991) The Drosophila Broad-Complex encodes a family of related proteins containing zinc fingers. Genetics 129: 385–397. 1743483 10.1093/genetics/129.2.385 PMC1204631

[18] Karim FD, Guild GM, Thummel CS (1993) The Drosophila Broad-Complex plays a key role in controlling ecdysone-regulated gene expression at the onset of metamorphosis. Development 118: 977–988. 8076529 10.1242/dev.118.3.977

[19] Consoulas C, Levine RB, Restifo LL (2005) The steroid hormone-regulated gene Broad Complex is required for dendritic growth of motoneurons during metamorphosis of Drosophila. J Comp Neurol 485: 321–337. 10.1002/cne.20499 15803508

[20] Mirth CK, Truman JW, Riddiford LM (2009) The ecdysone receptor controls the post-critical weight switch to nutrition-independent differentiation in Drosophila wing imaginal discs. Development 136: 2345–2353. 10.1242/dev.032672 19515698 PMC2729347

[21] Schubiger M, Carre C, Antoniewski C, Truman JW (2005) Ligand-dependent de-repression via EcR/USP acts as a gate to coordinate the differentiation of sensory neurons in the Drosophila wing. Development 132: 5239–5248. 10.1242/dev.02093 16267093

[22] Zeng X, Hou SX (2012) Broad relays hormone signals to regulate stem cell differentiation in Drosophila midgut during metamorphosis. Development 139: 3917–3925. 10.1242/dev.083030 23048182 PMC3472594

[23] Jiang C, Lamblin AF, Steller H, Thummel CS (2000) A steroid-triggered transcriptional hierarchy controls salivary gland cell death during Drosophila metamorphosis. Mol Cell 5: 445–455. 10.1016/S1097-2765(00)80439-6 10882130

[24] Kai T, Spradling A (2004) Differentiating germ cells can revert into functional stem cells in Drosophila melanogaster ovaries. Nature 428: 564–569. 10.1038/nature02436 15024390

[25] Gilboa L, Lehmann R (2004) Repression of primordial germ cell differentiation parallels germ line stem cell maintenance. Curr Biol 14: 981–986. 10.1016/j.cub.2004.05.049 15182671

[26] Lantz V, Chang JS, Horabin JI, Bopp D, Schedl P (1994) The Drosophila orb RNA-binding protein is required for the formation of the egg chamber and establishment of polarity. Genes Dev 8: 598–613. 10.1101/gad.8.5.598 7523244

[27] Forbes AJ, Spradling AC, Ingham PW, Lin H (1996) The role of segment polarity genes during early oogenesis in Drosophila. Development 122: 3283–3294. 8898240 10.1242/dev.122.10.3283

[28] Hodin J, Riddiford LM (2000) Different mechanisms underlie phenotypic plasticity and interspecific variation for a reproductive character in drosophilids (Insecta: Diptera). Evolution 54: 1638–1653. 10.1111/j.0014-3820.2000.tb00708.x 11108591

[29] Morin X, Daneman R, Zavortink M, Chia W (2001) A protein trap strategy to detect GFP-tagged proteins expressed from their endogenous loci in Drosophila. Proc Natl Acad Sci U S A 98: 15050–15055. 10.1073/pnas.261408198 11742088 PMC64981

[30] Riddiford LM, Cherbas P, Truman JW (2000) Ecdysone receptors and their biological actions. Vitam Horm 60: 1–73. 10.1016/S0083-6729(00)60016-X 11037621

[31] Hodin J, Riddiford LM (1998) The ecdysone receptor and ultraspiracle regulate the timing and progression of ovarian morphogenesis during Drosophila metamorphosis. Dev Genes Evol 208: 304–317. 10.1007/s004270050186 9716721

[32] Konig A, Yatsenko AS, Weiss M, Shcherbata HR (2011) Ecdysteroids affect Drosophila ovarian stem cell niche formation and early germline differentiation. EMBO J 30: 1549–1562. 10.1038/emboj.2011.73 21423150 PMC3102283

[33] Li MA, Alls JD, Avancini RM, Koo K, Godt D (2003) The large Maf factor Traffic Jam controls gonad morphogenesis in Drosophila. Nat Cell Biol 5: 994–1000. 10.1038/ncb1058 14578908

[34] Ashburner M, Chihara C, Meltzer P, Richards G (1974) Temporal control of puffing activity in polytene chromosomes. Cold Spring Harb Symp Quant Biol 38: 655–662. 10.1101/SQB.1974.038.01.070 4208797

[35] Bernardo TJ, Dubrovskaya VA, Xie X, Dubrovsky EB (2014) A view through a chromatin loop: insights into the ecdysone activation of early genes in Drosophila. Nucleic Acids Res 42: 10409–10424. 10.1093/nar/gku754 25143532 PMC4176353

[36] Contrino S, Smith RN, Butano D, Carr A, Hu F, et al (2012) modMine: flexible access to modENCODE data. Nucleic Acids Research 40: D1082–D1088. 10.1093/nar/gkr921 22080565 PMC3245176

[37] Gauhar Z, Sun LV, Hua S, Mason CE, Fuchs F, et al (2009) Genomic mapping of binding regions for the Ecdysone receptor protein complex. Genome Res 19: 1006–1013. 10.1101/gr.081349.108 19237466 PMC2694480

[38] Ray P, De S, Mitra A, Bezstarosti K, Demmers JA, et al (2016) Combgap contributes to recruitment of Polycomb group proteins in Drosophila. Proc Natl Acad Sci U S A 113: 3826–3831. 10.1073/pnas.1520926113 27001825 PMC4833261

[39] Li G, Ruan X, Auerbach RK, Sandhu KS, Zheng M, et al (2012) Extensive promoter-centered chromatin interactions provide a topological basis for transcription regulation. Cell 148: 84–98. 10.1016/j.cell.2011.12.014 22265404 PMC3339270

[40] Wood AM, Van Bortle K, Ramos E, Takenaka N, Rohrbaugh M, et al (2011) Regulation of chromatin organization and inducible gene expression by a Drosophila insulator. Mol Cell 44: 29–38. 10.1016/j.molcel.2011.07.035 21981916 PMC3190163

[41] Moeller ME, Danielsen ET, Herder R, O'Connor MB, Rewitz KF (2013) Dynamic feedback circuits function as a switch for shaping a maturation-inducing steroid pulse in Drosophila. Development 140: 4730–4739. 10.1242/dev.099739 24173800 PMC3833430

[42] Ou Q, Magico A, King-Jones K (2011) Nuclear receptor DHR4 controls the timing of steroid hormone pulses during Drosophila development. PLoS Biol 9: e1001160 10.1371/journal.pbio.1001160 21980261 PMC3181225

[43] Maimon I, Gilboa L (2011) Dissection and staining of Drosophila larval ovaries. J Vis Exp. 10.3791/2537 21610675 PMC3197100

[44] Mugat B, Brodu V, Kejzlarova-Lepesant J, Antoniewski C, Bayer CA, et al (2000) Dynamic expression of broad-complex isoforms mediates temporal control of an ecdysteroid target gene at the onset of Drosophila metamorphosis. Dev Biol 227: 104–117. 10.1006/dbio.2000.9879 11076680

[45] Simonis M, Kooren J, de Laat W (2007) An evaluation of 3C-based methods to capture DNA interactions. Nat Methods 4: 895–901. 10.1038/nmeth1114 17971780

[46] van de Werken HJ, Landan G, Holwerda SJ, Hoichman M, Klous P, et al (2012) Robust 4C-seq data analysis to screen for regulatory DNA interactions. Nat Methods 9: 969–972. 10.1038/nmeth.2173 22961246

[47] Pile LA, Wassarman DA (2002) Localizing transcription factors on chromatin by immunofluorescence. Methods 26: 3–9. 10.1016/S1046-2023(02)00002-6 12054899

