# Supplementary figures and images for: Combgap Promotes Ovarian Niche Development and Chromatin Association of EcR-Binding Regions in BR-C
Source: PLoS Genet. 2016 Nov 15;12(11):e1006330. doi: 10.1371/journal.pgen.1006330 (PMC5147775; doi:10.1371/journal.pgen.1006330)

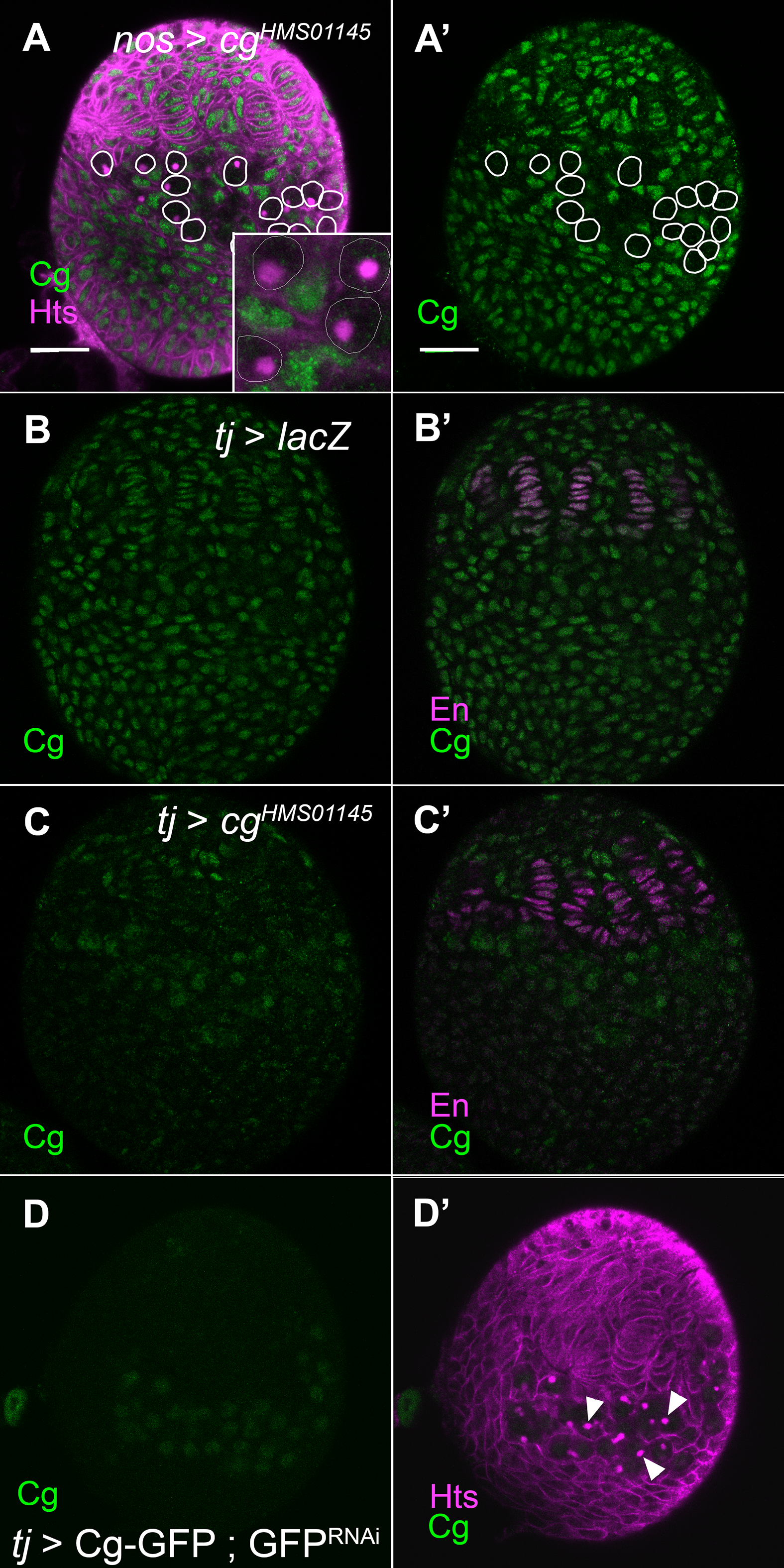

Supplement: S1 Fig — In all panels, anti-Cg is in green. (A) Anti-Hts (magenta) outlines somatic cells and fusomes within PGCs. The germline driver nos-Gal4 removes Cg specifically from PGCs (outlined in A, A’). Fusomes in PGCs remain spherical (inset) showing germ cells have not differentiated into cysts. (B, B’) Control LL3 ovaries showing Cg in all nuclei and well-formed TFs (anti-En, magenta). (C, C’) the somatic driver tj-Gal4 drives both DicerII and an RNAi construct directed against Cg. Cg is still apparent in germ cell nuclei and in anterior nuclei where tj-Gal4 is not expressed. However, remnants of Cg protein can still be seen in nuclei throughout the ovary. (D, D’) tj-Gal4 drives DicerII and GFPRNAi in Cg-GFP ovaries. Cg is still expressed in PGCs, while very little Cg can be observed in somatic nuclei. PGCs do not differentiate and carry spherical fusomes (D’, arrowheads, anti-Hts, magenta). (TIF) [file pgen.1006330.s001.tif]

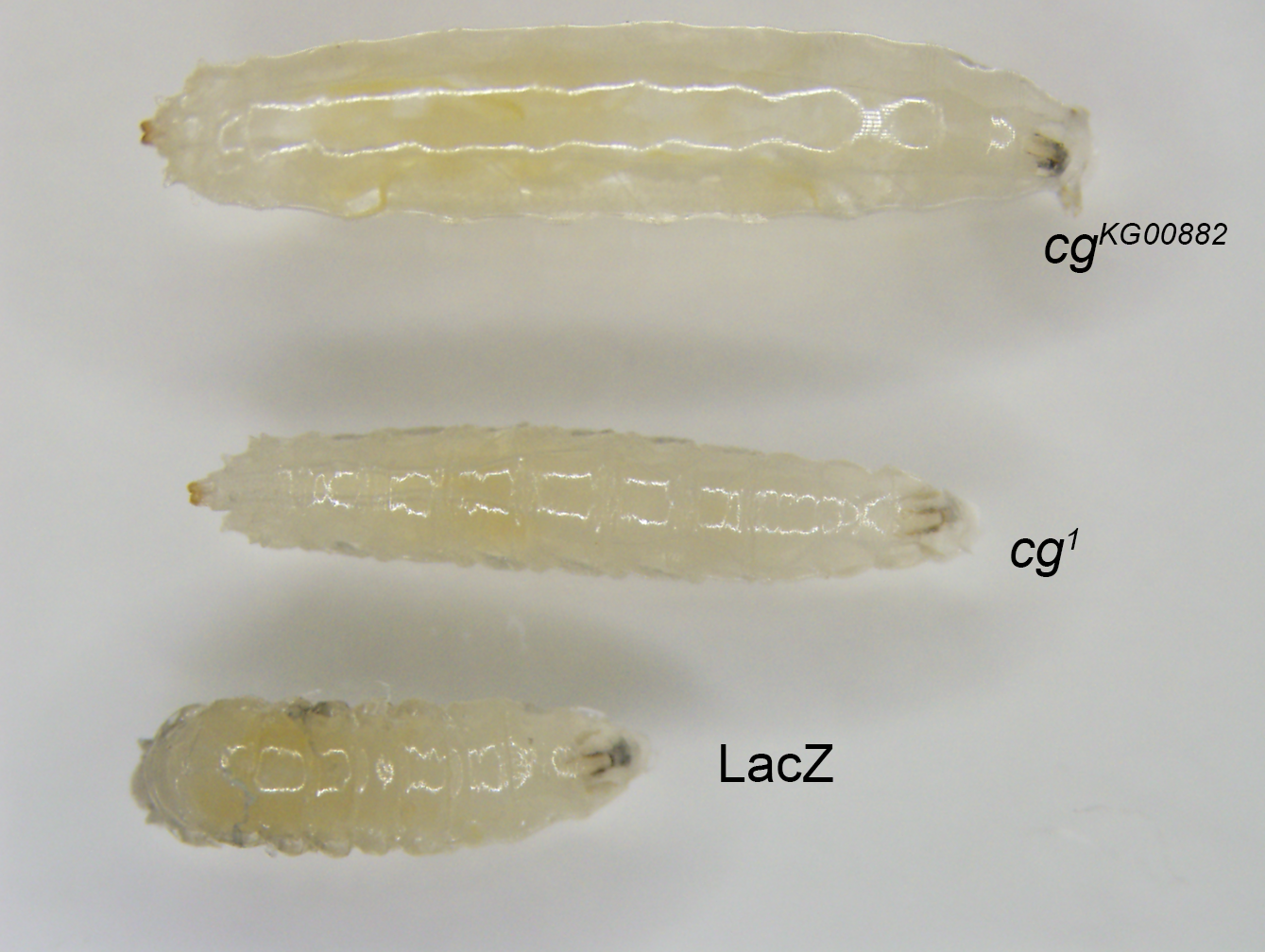

Supplement: S2 Fig — cg1 or cgKG00882 alleles were balanced on the attached chromosome SM6a-TM6,Tb, such that homozygous larvae were easily recognized by their lack of Tb phenotype. A control LacZ larva at the wandering stage, 5 days after egg laying is shown for comparison. cg1 or cgKG00882 giant larvae were collected from a bottle 8 days after egg laying. The mutants were still at the larval stage, while their heterozygote siblings were already pupae. (TIF) [file pgen.1006330.s002.tif]

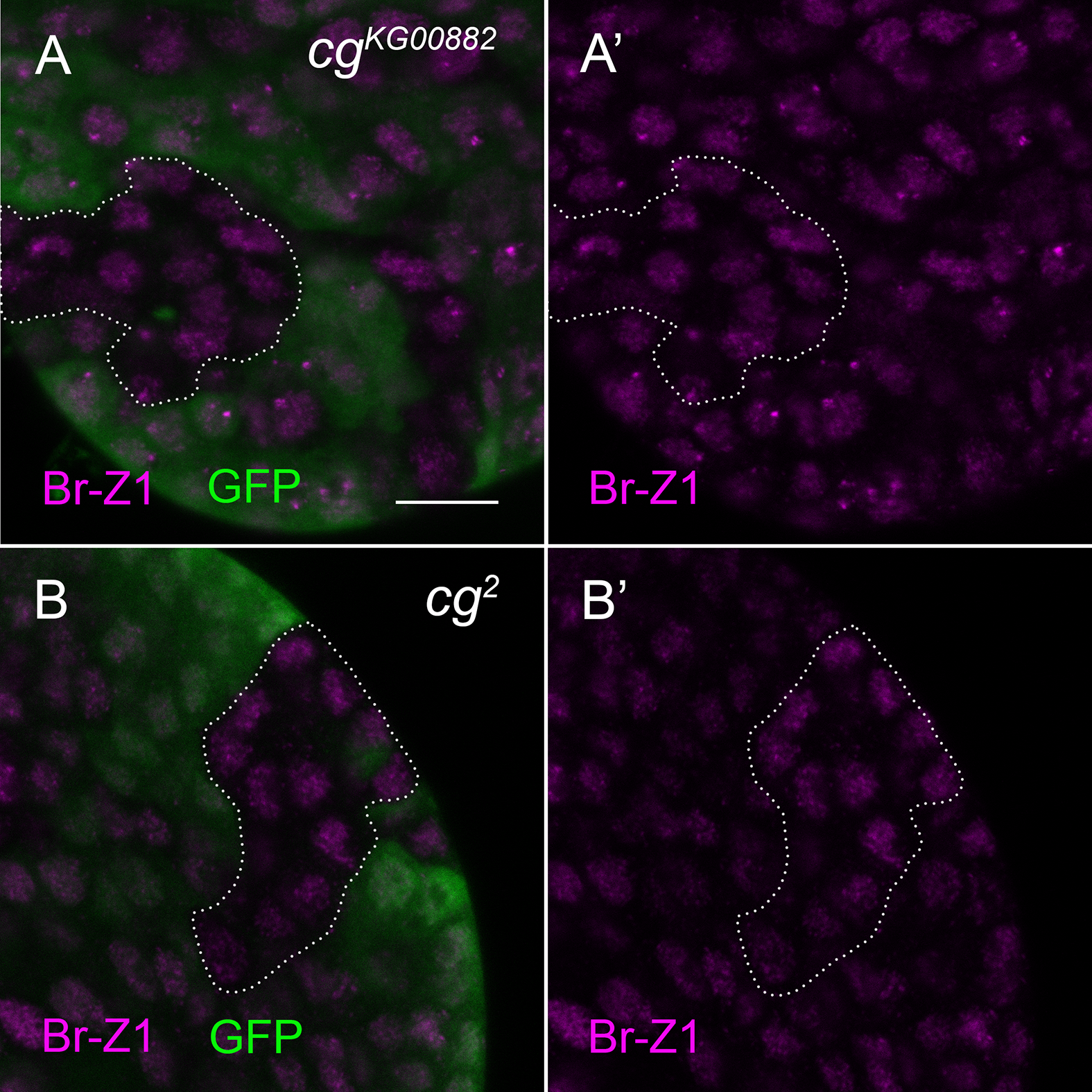

Supplement: S3 Fig — In all panels, Anti-Br-Z1 is in magenta and anti-GFP is in green. Mutant cells lack GFP and are outlined. Similar levels of Br-Z1 protein are present in cgKG00882 (A, A’) or cg2 (B, B’) mutant clones as compared with their WT neighbors. Bar is 10 μm for all panels. (TIF) [file pgen.1006330.s003.tif]

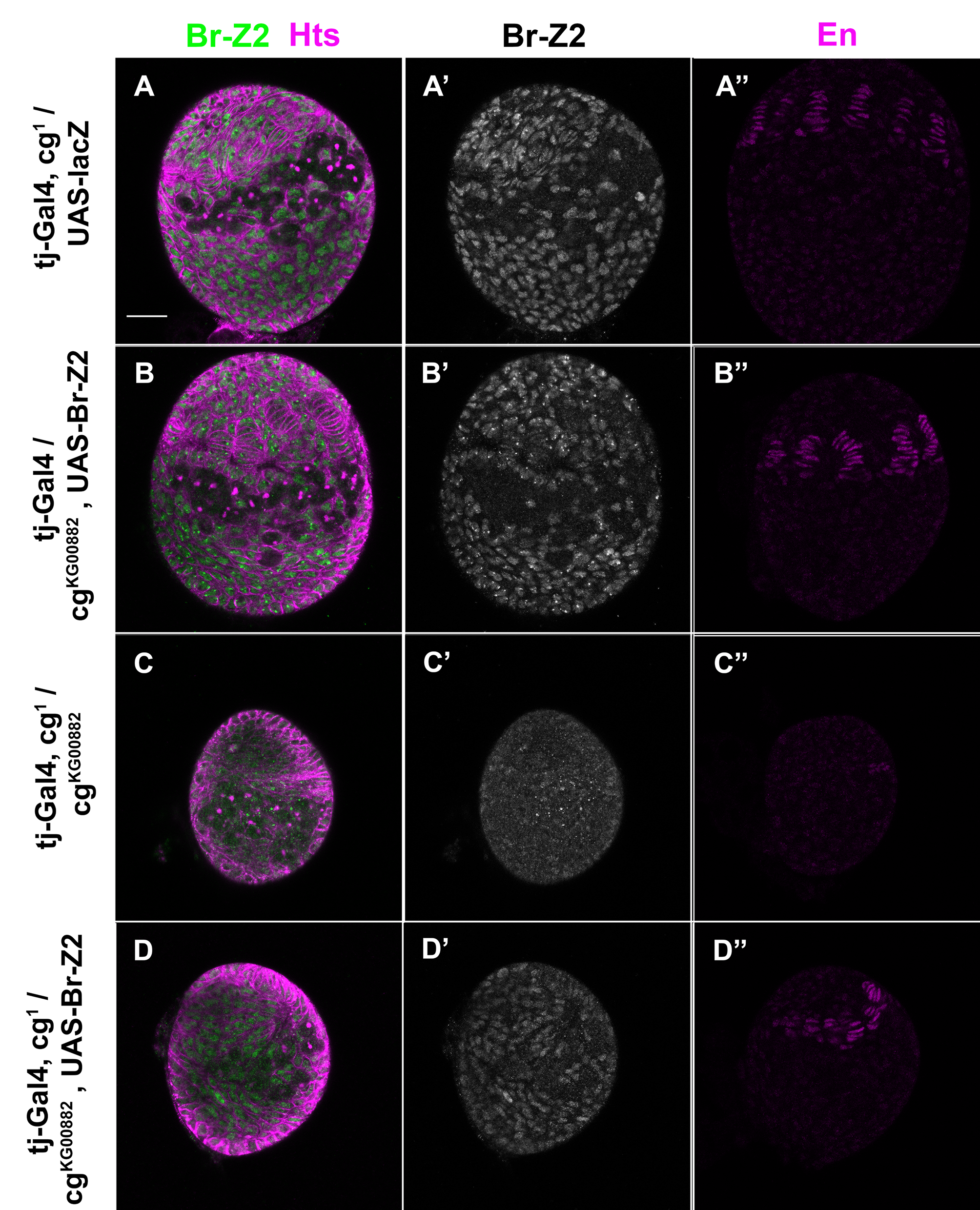

Supplement: S4 Fig — Anti-Br-Z2 is in green or grey. Anti-Hts outlines somatic cells and labels fusomes within PGCs (magenta in A, B, C, D). Anti-En (magenta in A”, B”, C”, D”) labels TFs. (A, A’, A”) control ovaries, showing normal Br-Z2 expression and normal TFs. (B, B’, B”) Over-expression of Br-Z2 in a WT background, showing that over-expressing this protein does not result in a large BR-Z2 increase above WT levels and does not change normal ovarian development. (C, C’, C”) cg-mutant ovaries showing reduced size, severe defects in TF formation, no posterior somatic cells, and very little expression of Br-Z2. (D, D’, D”) cg-mutant ovaries over-expressing Br-Z2, ovaries increase in size compared to cg-mutants (compare to S4C), contain more TFs (compare to S4C”, Table 1), and have a sizable population of posterior somatic cells. However, rescued ovaries do not reach the advanced developmental stage of WT ovaries (compare to S4A). (TIF) [file pgen.1006330.s004.tif]

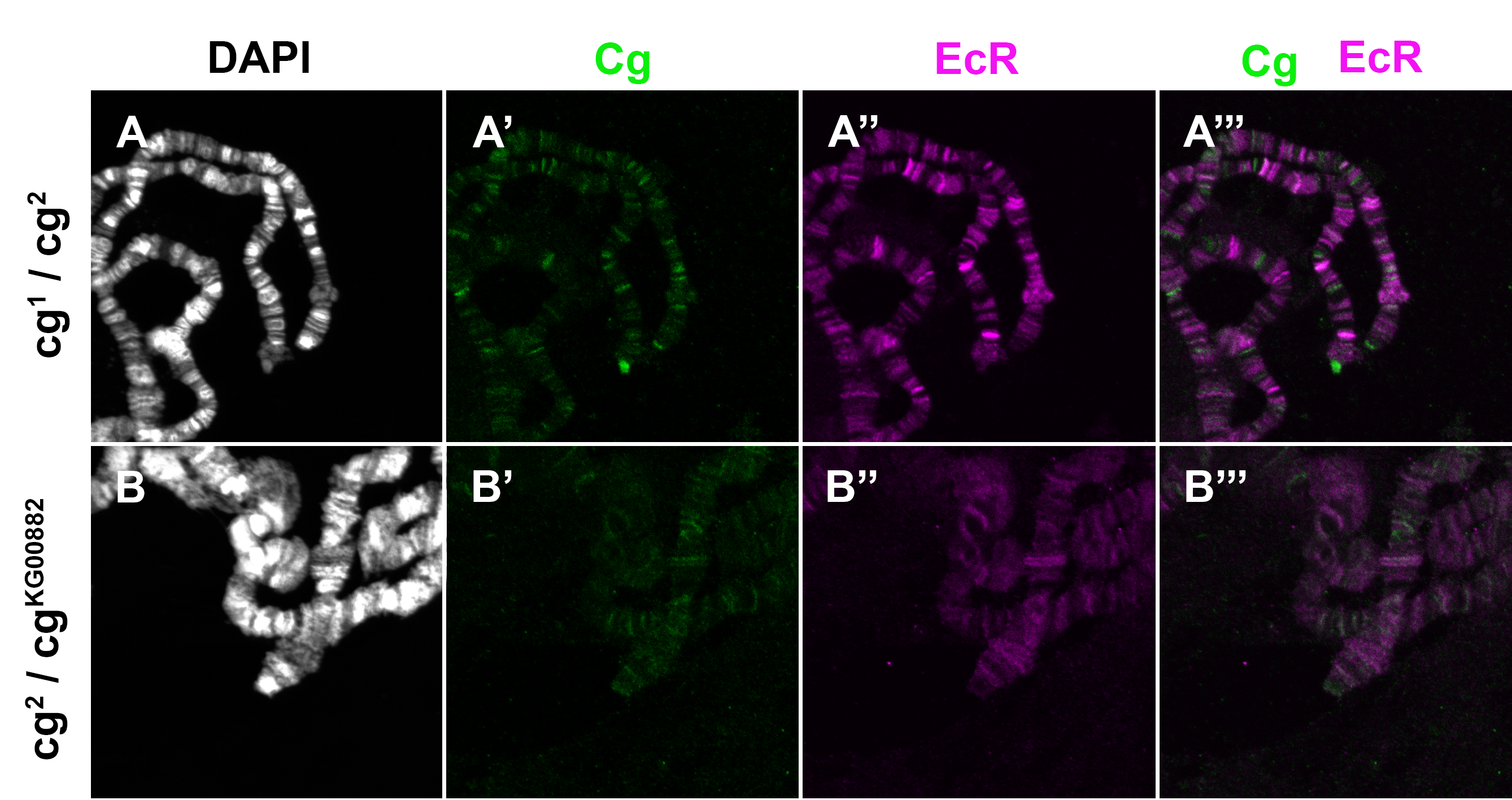

Supplement: S5 Fig — Spreads of polytene chromosomes from salivary glands were stained with Dapi (white), anti-Cg (green) and anti-EcR (magenta). Glands were stained and imaged using the same confocal settings and on the same day as those of WT (Compare to Fig 6). Cg staining is weaker than WT in the cg1/cg2 (A-A”‘) and cg2/cgKG00882 (B-B”‘). The weakening in EcR staining correlates with the level of Cg protein remaining on the polytene chromosomes. (TIF) [file pgen.1006330.s005.tif]

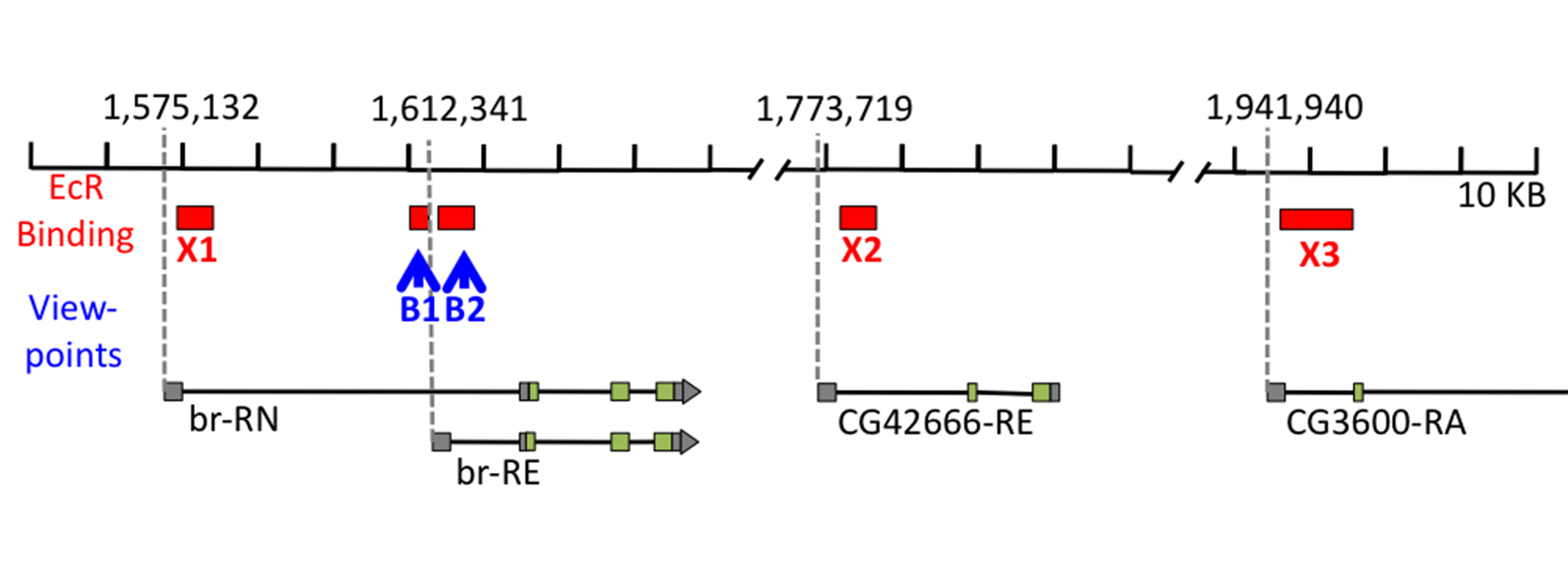

Supplement: S6 Fig — Gene structure and genomic positions are according to release 6. The locations of the EcR-enriched binding regions are in red, and the viewpoints are in blue. (TIF) [file pgen.1006330.s006.tif]

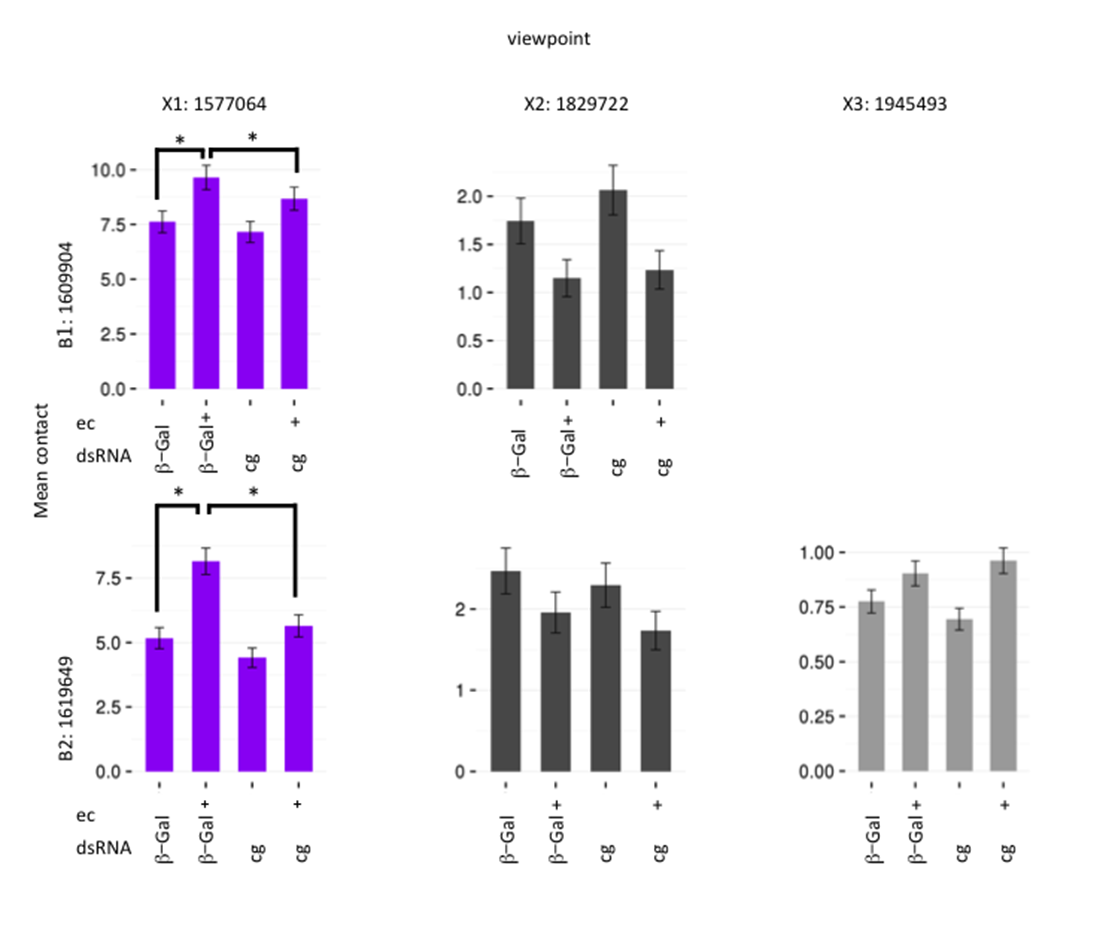

Supplement: S7 Fig — Bar plots showing the mean contact of the viewpoints (X1, X2, X3) with B1 and B2. For windows B1, B2, interaction with X1 30 fragments per each and 200 fragments for X3 interaction with B1, B2. While the number of contacts between B1/B2 and X2/X3 is low, the tendencies of interactions remain similar in the reciprocal 4C. *P<0.001 (Chi-Square pair-wise test). (TIF) [file pgen.1006330.s007.tif]

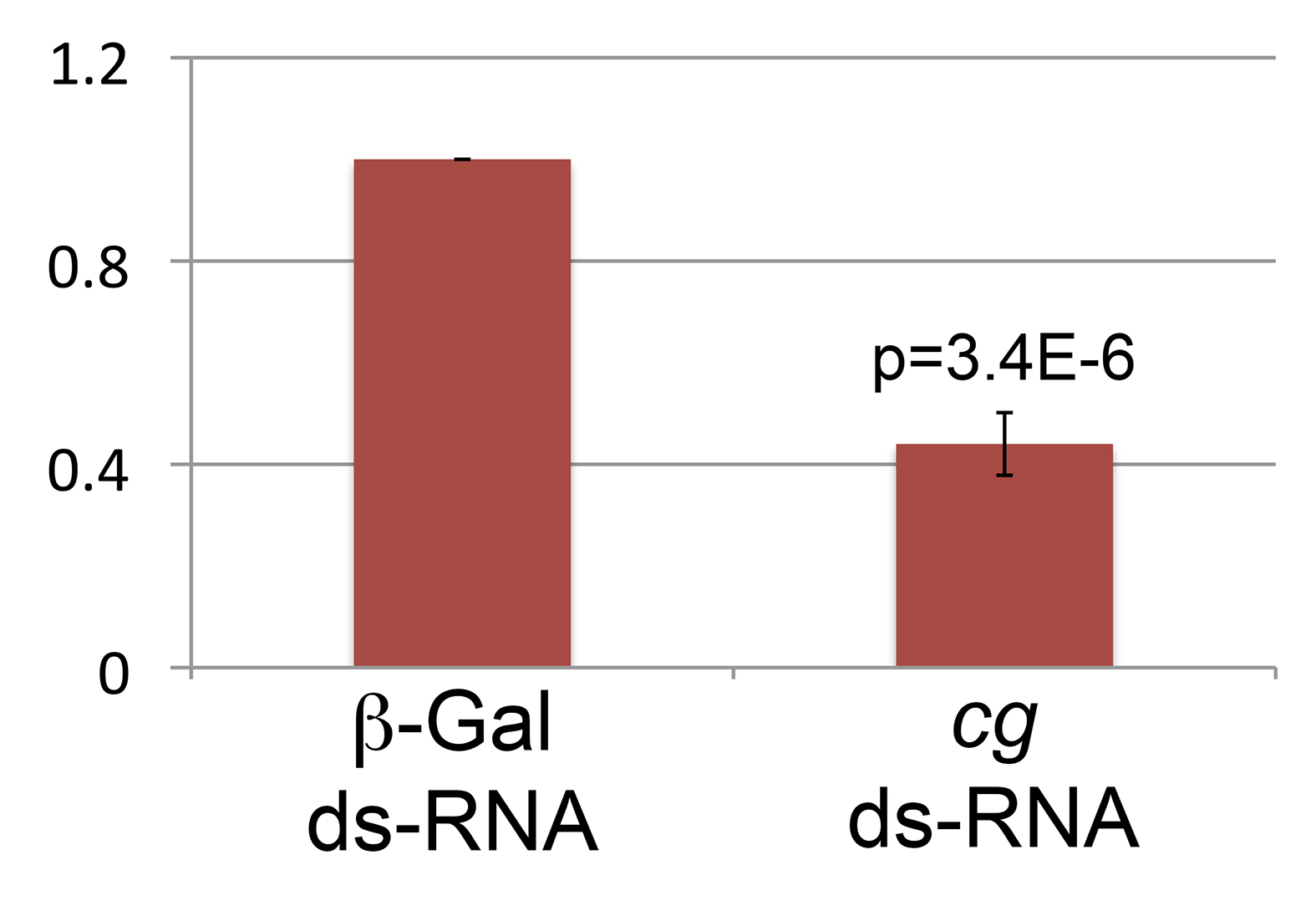

Supplement: S8 Fig — Cells were treated with either control, β-Gal ds-RNA or with cg ds-RNA. cg mRNA levels were measured by qPCR. The data presented is derived from 5 biological repeats. (TIF) [file pgen.1006330.s008.tif]

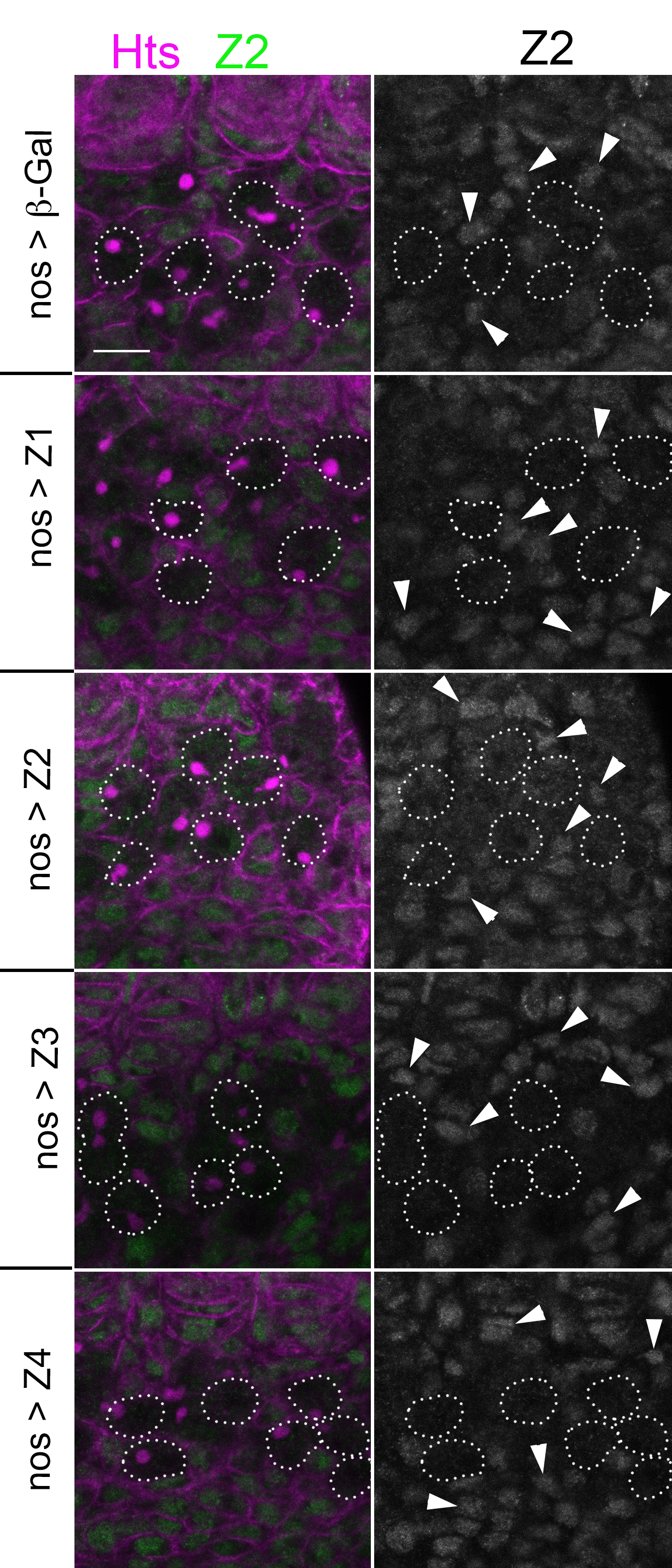

Supplement: S9 Fig — Ovaries were stained with Anti-Br-Z2 (green or white) and with anti-Hts (magenta). PGCs are outlined. Control ovaries (nos-Gal4>b-Gal) show anti-Z2 expression only in somatic cells, but not in PGCs. Each of the four BR-Z isoforms was expressed in germ cells using the driver nos-Gal4. The BR-Z2 antibody stains germ cells only upon expression of BR-Z2, attesting to the specificity of the antibody. (TIF) [file pgen.1006330.s009.tif]

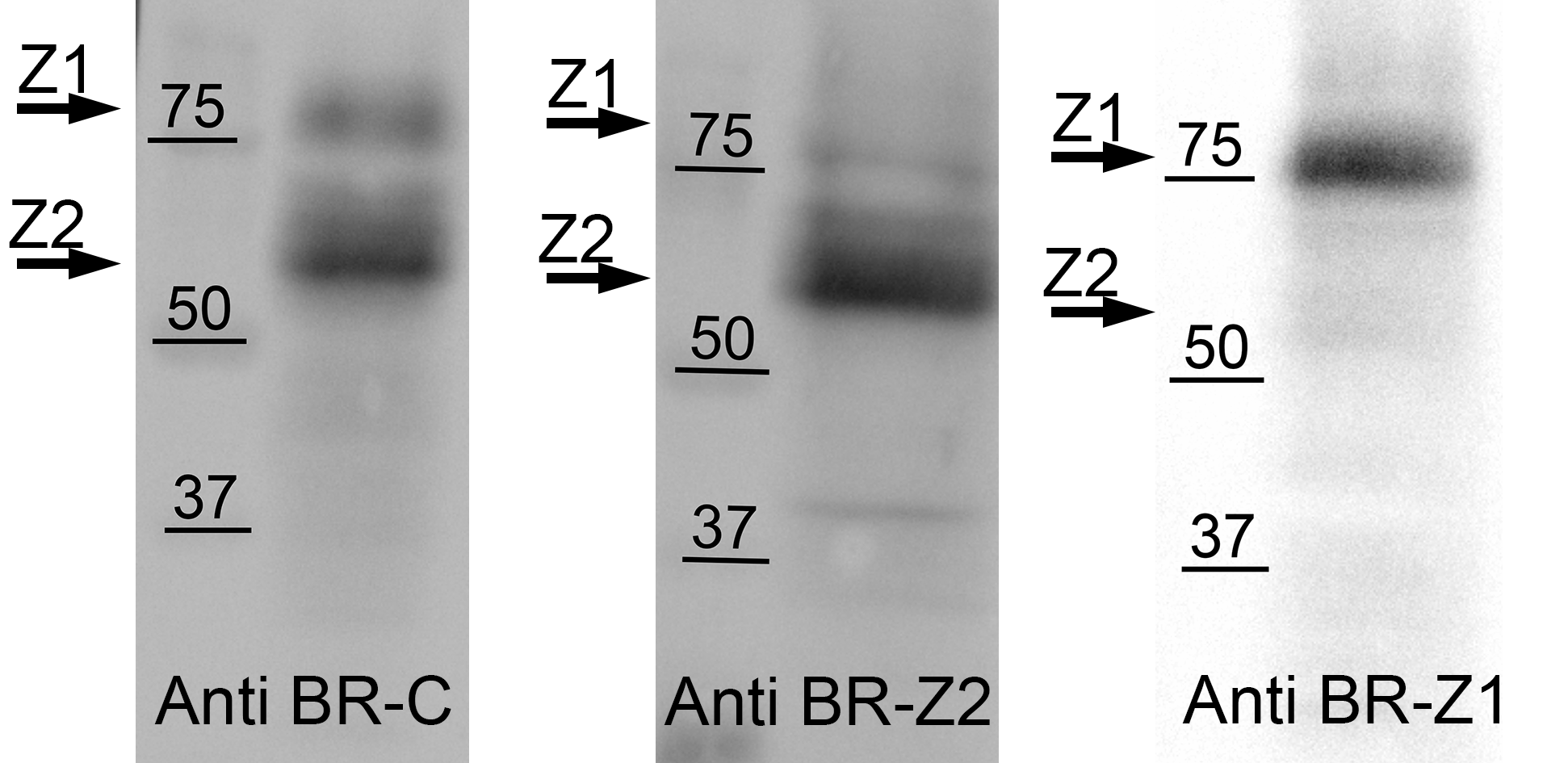

Supplement: S10 Fig — Western Blot analysis of imaginal discs using various anti-Br antibodies. Anti-BR-C recognizes the two major BR-C isoforms that are expressed in the discs (BR-Z1 and BR-Z2, indicated). Anti-BR-Z2 and anti-BR-Z1 each recognizes an individual isoform. (TIF) [file pgen.1006330.s010.tif]
